# Factors influencing the implementation of a guideline for re-engagement in HIV care in primary care settings in Johannesburg, South Africa: A qualitative study

**DOI:** 10.1371/journal.pgph.0003765

**Published:** 2024-10-30

**Authors:** Ndinda Makina-Zimalirana, Lynne Susan Wilkinson, Anna Grimsrud, Natasha Davies, Chipo Mutyambizi, Anele Jiyane, Fezile Buthelezi, Kate Rees

**Affiliations:** 1 Anova Health Institute, Johannesburg, South Africa; 2 International AIDS Society, Cape Town, South Africa; 3 Department of Community Health, School of Public Health, University of Witwatersrand, Johannesburg, South Africa; McGill University, CANADA

## Abstract

Re-engagement, which involves bringing individuals who have fallen out of HIV care back into treatment, is important in the ongoing care of individuals with HIV, especially in regions with high prevalence and resource limitations. Despite extensive treatment programs, a significant number of people living with HIV in South Africa disengage from care due to different barriers. To address this, the South African Department of Health (DoH) introduced guidelines to support re-engagement. However, while there is a lot of research on factors leading to disengagement, there is a gap in understanding effective strategies for retaining those who re-engage. The objective of this study is to understand the barriers and facilitators influencing the adoption and scalability of strategies for re-engagement in HIV care. Anova Health Institute, in collaboration with the Johannesburg district DoH, launched the Re-engagement Initiative. This initiative aimed to help healthcare providers better understand and implement re-engagement guidelines through capacity-building, clinical decision support tools, mentorship, and data collection. We conducted a qualitative study across nine primary care facilities in Johannesburg to investigate the perspectives of implementing providers. Data collection involved in-depth interviews using semi-structured guides. The Consolidated Framework for Implementation Research (CFIR) was used to analyse factors influencing implementation. Our study identified several factors affecting the implementation of intervention supporting re-engagement guidelines. Leadership was important for driving organizational change, creating the necessary tension for change, and prioritizing the intervention. Knowledge and beliefs about the intervention were also significant; while most providers understood the initiative’s objectives and tools, negative attitudes among some hindered adoption. Empathy for client disengagement motivated some providers, while others did not share this understanding. The belief that job aides and re-engagement forms promoted standardized care and improved documentation was a factor in supporting the initiative. Additionally, the alignment of the intervention with existing guidelines, facility plans, and goals influenced its success and sustainability. Our findings offer valuable insights into the opportunities and challenges of implementing intervention to support re-engagement guidelines. They emphasize the need to address negative provider attitudes, foster engaged leadership, and integrate initiatives with broader HIV care program and facility workflows. These insights are important for the adoption and implementation of similar guidelines in similar settings.

## Introduction

Re-engagement, which involves bringing individuals who have fallen out of HIV care back into treatment, plays an important role in the cyclical HIV care continuum [1], particularly in low- and middle-income countries with high HIV prevalence [2]. In these settings, individuals often encounter obstacles, both structural and personal, that lead to disengagement from care [2, 3]. Consequently, there has been a notable shift towards more individuals re-engaging than newly initiating treatment, particularly in eastern and southern Africa, [4]. South Africa boasts the largest HIV treatment program globally, providing antiretroviral therapy (ART) to over 5.7 million people by the end of 2022 [5]. Despite this extensive program, an estimated 2.5 million people living with HIV in South Africa remain untreated [6], which includes individuals who started but subsequently discontinued treatment. A systematic review suggests that between 20% and 50% of ART clients, with a probable minimum of 30%, who present for ART are re-initiators [4].

To respond to this reality, in 2020, the South African Department of Health (DoH) developed guidance aligned with that of the WHO [7, 8], emphasizing the identification and tracking of people who have missed appointments, and a differentiated service delivery (DSD) approach to support re-engagement [9]. Depending on clinical condition at re-engagement and length of treatment interruption, re-engaging clients could access multi-month dispensing (MMD) and less-intensive DSD models (known in South Africa as “repeat prescription collection strategies” (RPCs)) to facilitate sustained re-engagement.

There has been extensive research into the factors that lead to disengagement [10–12], but there is still a notable gap in our understanding of successful approaches to retain individuals who re-engage [1, 12, 13]. While it is important to identify interventions that can improve the likelihood of a person not disengaging again, it is equally important to understand how these interventions should be effectively implemented in HIV programs. Multiple strategies targeting healthcare professionals, including the provision of written information (clinical decision support), capacity-building initiatives, and audit and feedback mechanisms, are commonly employed to facilitate the implementation of guidelines within clinical settings [14, 15]. These approaches hold great potential for enhancing client-centred care delivered by clinicians and other healthcare providers. For example, clinical decision support tools can strengthen knowledge, offer decision-making algorithms or strategies [16]. When integrated into shared decision-making processes, clinical decision support tools can provide clients with vital information, aid in clarifying their preferences, and prepare them for making informed decisions and understanding management plans and timelines [15]. However, such tools are often not seamlessly integrated into routine care [16, 17] and identifying factors influencing successful implementation is critical for healthcare systems.

To ensure that healthcare providers involved in HIV care were well-informed about and effectively implemented re-engagement guidelines, Anova Health Institute, in collaboration with the Johannesburg District DoH, implemented the Re-engagement Initiative. This initiative included a capacity-building component, development of clinical decision support tools outlining national guidance, site visits to provide mentorship and feedback, and data collection for monitoring and evaluation. The Initiative aimed to facilitate sustained re-engagement. We conducted a mixed method evaluation using quantitative and qualitative methods, with the quantitative data published elsewhere [18]. This study, based on the qualitative data, investigated the perspectives of implementing providers, focusing on identifying the barriers and facilitators influencing adoption and scalability. Our aim was to gather insights to inform future implementation of re-engagement interventions.

## Methods

### The intervention: Re-engagement initiative

The initiative was a multifaceted approach to implementing an existing guideline [9], comprised of training, clinical decision tools (referred to as job aides), and a combination clinical decision and data collection tool (referred to as re-engagement forms). It was designed to promote a differentiated approach to individuals re-engaging in HIV care after missing a scheduled appointment, including clinical management, counselling, visit frequency, and access to South Africa’s less-intensive DSD models options. Table 1 describes the initiative’s key components, and categorises them according to the Expert Recommendations for Implementing Change (ERIC) implementation strategies framework [19]. Table 2 outlines the roles and responsibilities for nurses, retention counsellors, and data clerks participating in the initiative. The tools can be found in S1 File.

**Table 1:** Components of re-engagement initiative.

Component	Description	ERIC Implementation Strategy
**Leadership engagement**	Introduce the intervention to facility leadership, and obtain support from district management, cascading to facility managers.	*Mandate change*: Have leadership declare the priority of the innovation and their determination to have it implemented
**Training**	In-service training covered various aspects of re-engagement, including objectives and procedures. Training was conducted using a train-the-trainer (i.e., cascading training to expand reach) approach.	*Use train-the-trainer strategies*: Train designated clinicians or organizations to train others in the clinical innovation. *Conduct ongoing training*: Plan for and conduct training in the clinical innovation in an ongoing way
**Job Aides**	Clinical decision tool designed to assist healthcare providers in re engagement tasks. These aides include checklists, flowcharts, and decision support tools. They served as quick references to ensure that healthcare professionals follow standardized procedures and protocols effectively. There was a job aide for admin clerks, one for clinicians, and one for counsellors.	*Develop educational materials*: Develop and format manuals, toolkits, and other supporting materials in ways that make it easier for stakeholders to learn about the innovation and for clinicians to learn how to deliver the clinical innovation.
**Re-engagement forms**	Documents used to systematically guide clinicians through the re-engagement consultation, and simultaneously gather information on re-engaging clients and the appropriate management plan. These forms were specifically designed to capture relevant data points, ensuring accurate and consistent data collection.	*Develop and organize quality monitoring systems*: Develop and organize systems and procedures that monitor clinical processes and/or outcomes for the purpose of quality assurance and improvement
**Site level support for implementation**	Involved on-site assistance and guidance provided by a dedicated nurse mentor, including feedback on clinician assessments and facility performance	*Provide clinical supervision*: Provide clinicians with ongoing supervision focusing on the innovation. *Provide local technical assistance*: Develop and use a system to deliver technical assistance focused on implementation issues using local personnel. *Audit and provide feedback*: Collect and summarize clinical performance data over a specified time period and give it to clinicians and administrators to monitor, evaluate, and modify provider behavior.

**Table 2:** Responsibilities.

Staff Category	Role
**Data clerk**	• Identify clients who had missed scheduled ART appointments. • Mark on register, indicating whether appointment missed by ≤ or >14 days. • Insert re-engagement form in client folder. • Support navigation to appropriate clinician queue.
**Clinician (nurse or doctor)**	• Conduct re-engagement clinical assessment using re-engagement form. • Determine and carry out follow-up plan, including visit schedule, MMD and (r)enrolment in less-intensive DSD models. • Dispense ART refill length as directed. • Confirm next location for ART refill collection with client. • Manage script submission for DSD models where appropriate.
**Adherence Counsellors**	• Support navigation to appropriate clinician queue. • Print/document missing lab results and place in folder. • Provide appropriate counselling sessions.

### Study design

This study employed a qualitative cross-sectional design. Qualitative data were collected through interviews.

#### Study setting

The study was conducted in nine primary care facilities in Johannesburg. Johannesburg is the economic epicentre of South Africa and has a population of 6 million. It is home to approximately 677,500 people living with HIV, with an adult HIV prevalence of 16.4% [20]. The initiative was implemented in 3 of the city’s 7 sub-districts. The facilities had an average monthly total remaining on ART ranging between 750 and 32 000 (DHIS,2023). Implementation sites were purposively selected based on the health facilities’ expressed interest, while ensuring a mix of both large and small healthcare facilities distributed across the three sub-districts. The implementation started with meetings to engage leadership at facility, then moved on to training the appropriate staff members.

### Data collection

Interviews were conducted by authors AJ and FB, highly experienced and well-trained female and male qualitative research facilitator, respectively. Purposive sampling was used to sample healthcare providers to ensure adequate representation across different facilities and staff roles. Healthcare providers were defined as: clinical providers (nurses or doctors), non-clinical staff (retention counsellors and data clerk) and managers. Potential participants were contacted telephonically to invite participation. Participants provided written informed consent. Interviews commenced with an introduction of the research facilitator. Participants were encouraged to share their honest perceptions, whether positive or negative, and were informed about the overall goals of the study. The interviews were conducted in person in a private space at the participants’ healthcare facility and were audio recorded. The audio interviews were transcribed. The transcripts were not returned to participants for comment or verification. No repeat interviews were conducted in this study. Data saturation was not discussed because the study was designed to collect diverse perspectives. Multiple and diverse data sources, including various participants and settings, were used to enrich the richness and representativeness of the data. The study employed purposive sampling to gather insights from different perspectives.

The interviews were conducted using semi-structured guide adapted from interview guide used in previous study [21]. The guide was tailored to align with the study’s objectives. Questions were not pretested before data collection. The guide had two sections and covered several topics. The first section aimed to gain an understanding of participants’ perceptions of re-engagement, support available to the healthcare providers, and the challenges and successes of re-engaging clients. The second section focused on participants’ experiences with implementing the Re-engagement Initiative, exploring aspects such as training, utility/acceptability of the job aides, views on the relative advantages of the initiative, resources, availability of provider support, supervision, the level of "buy-in" or political will to execute the initiative, and recommendations to improve implementation. The questions were tailored to role categories to elicit context-specific information (S2 File).

### Framework

The Consolidated Framework for Implementation Research (CFIR) serves as a valuable framework for understanding the processes involved in implementing an intervention and understanding how interventions become feasible and integrated into everyday work [22]. CFIR focuses on the operational task’s individuals undertake to put a set of practices into action. Moreover, CFIR aids in generating clear recommendations for future implementation efforts It was selected to highlight the experiences of staff in making the initiative work in practice. We anticipated that CFIR could provide insights into the factors conducive to the adoption and sustainability of the intervention. The framework comprises 5 main constructs, as outlined below.

1. Intervention Characteristics: Stakeholder Perspectives
  This pertains to how stakeholders perceive the intervention, including its adaptability and how it compares in terms of advantages over existing interventions.
2. Outer Setting: External Influences
  This considers the influence of external factors, such as policies at other organizations, which may have an impact on decisions made during the implementation process. This could include factors like client needs and available resources.
3. Inner Setting: Organizational Attributes
  This encompasses the attributes within the organization that may influence the implementation process. For instance, it includes the organizational climate for implementation.
4. Characteristics of Individuals: Personal Attributes
  This focuses on the attributes of individuals involved in the intervention and how these attributes may influence the implementation process. This includes personal traits, knowledge, beliefs, and how individuals handle the complexity of the intervention.
5. Process: Implementation Methods
  This refers to the methods employed during the implementation of the intervention. It includes strategies like engaging local champions to support the process.

### Analysis

Transcription files were uploaded into NVivo 12 software for qualitative data management and analysis.

We conducted thematic analysis following established principles [23] to extract meaningful insights from the transcripts. Our study involved a coding and analytic approach with three researchers (NMZ, FB and AJ), combining elements of deductive and inductive methods. NMZ engaged in multiple readings of the transcripts to develop a deep familiarity with the content. NMZ initially derived deductive codes from the existing literature and CFIR framework on providers’ perceptions on implementation of HIV interventions, providing a structured foundation for our analysis. Subsequently, NMZ transformed the content into condensed meaning units and applied open coding to identify additional key codes. These codes were then systematically organized under sub-categories, after which they were collectively reviewed by NMZ, FB, and AJ to reach consensus. The sub-categories were further aggregated into broader categories, and the relationships among these categories were scrutinized within the framework of the CFIR. Not all CFIR elements were explicitly addressed during the interviews, as the interview guide had an open-ended nature.

### Ethics approval and participation consent

The study received ethical approval from HSRC REC (3/22/08/18) and the Johannesburg District Research Committee. Informed written consent was obtained from all participants before the interviews. Participants were assured that their identity would not be disclosed. Additionally, the consent included permission for the use of direct quotes in research reports or publications, provided that no identifying information would be disclosed. Participants were free to withdraw from the research process at any time and without any explanation or negative consequences.

## Results

### Participant characteristics

Data was collected from 30 November 2022 to 6 January 2023 in nine healthcare facilities. Each interview lasted between 30 to 60 minutes. A total of 22 healthcare providers participated in the interviews. The sample included 10 nurses, 6 retention counsellors, 5 data clerks, and 1 facility manager. Of the participants, 17 were women and 5 were men, reflecting the gender distribution commonly observed in healthcare roles in the region. Participants varied in age and years of service, with an average of three and a half years of experience in HIV clinics, and their experience ranged from one to ten years. From the perspective of healthcare providers, key implementation themes emerged within the CFIR domains:

#### Characteristics of individuals involved in intervention delivery

*Knowledge and beliefs about the initiative*. Most providers demonstrated good understanding of re-engagement in general and the initiative tools and job aides in particular. Additionally, most healthcare providers had strong personal beliefs on the importance of re-engaging clients into care. Participants recognized and empathized with the reasons for client disengagement, acknowledging the complexity of this issue, often involving interconnected barriers. Healthcare providers, especially retention counsellors, were sympathetic, highlighting that clients typically do not plan to disengage from care; instead, various life demands and circumstances, like providing for their families and managing other unrelated health issues, make it challenging for them to stay engaged.

*There are patients that are lost to follow due to not having a steady income, the ability to meet basic needs (food and transport money to come to the facility).* ***Retention Counsellor***

Furthermore, all cadres of healthcare providers recognised that unplanned travel and the lack of flexibility of clinic services sometimes made it impossible to attend appointments. The geographical distance to healthcare facilities, coupled with daily work commitments, was highlighted as an additional challenge that some clients go through:

*And also, the clinic doesn’t operate on weekends because they are at work during the week, some will say that they would have come if the clinic was open on weekends, they have reasons. And they will come to the clinic, and we will make them wait last in the queue because they have disengaged, and they end up getting bored and leaving the clinic without getting the assistance that they need.*

***Retention Counsellor***

This understanding of the complexities of client disengagement motivated some healthcare providers to commit to implementing the initiative.

However, some participants noted negative attitudes among other providers, expressing concerns about confrontational behaviours when clients missed their appointments which discouraged clients and affected re-engagement:

*There are those who never got fair treatment, I mean by the clinicians or any of the staff, some might feel like they are being judged.*

***Data Clerk***

There were healthcare providers who had negative attitudes towards re-engagement and believed it negatively impacts HIV care provision overall. Some felt that it disrupted services, as stated by this clinician:

*…my workload is still going up … it means now I have these 5 patients that I was not expecting and those that were scheduled for today, and those are the patients that complain.*

***Clinician***

Additionally, some clinicians showed a patronizing and blaming attitude towards clients, at times describing clients as "dishonest and uncommitted". There were instances where participants felt that clients did not give the same level of importance to their health as healthcare providers do. Some participants expressed the perspective that clients displayed a lack of commitment, suggesting that clients might be intentionally late or inattentive to their appointments, which was seen as a form of disregard for their own well-being.

*They [patients] don’t take it as important as the health providers. Because remember when someone wants to be late, they will be late which is being ignorant on their part.*

***Clinician***

Moreover, some clinicians expressed discomfort with prioritization of re-engaging clients, as it disadvantaged those who adhered to their appointment schedules. As one clinician lamented,

*But the thing is, the priority that the system says we must give them is the thing that makes me angry because these people decided to stop medication, now they’re coming back. They have liked to be seen first before those people who are here on the due date for their medication. That’s the thing that makes me angry. They said if they’re here, you must welcome them back with open hands. No, that is fine. But they must not be in front of those people who have been on treatment and not missing their medication.*

***Clinician***

Although the re-engagement forms were created to assist clinicians with developing a management plan, record keeping and standardising care, they also had the unintended consequence of clinicians using the forms to threaten clients experiencing challenges. For these clients, clinicians hoped that the documentation served as a wakeup call to address their adherence issues. This sentiment suggests a confrontational attitude in the interactions between clinicians and clients.

*But now I have the tool that I use to defend myself and the medication to say if you have missed 14 days, I must write you down and give the names to the superiors. Then you know why they do this. Ah, okay. I’ll never do it again. I’ll never do it again. And then again after that, you remove it. Yes. And paste it in their file clip it on the file and then say you, I’m in trouble.*

***Clinician***

*Self-efficacy*. Clinicians and data clerks indicated confidence in their ability to use the job aides and tools. This was an important factor facilitating delivery of the initiative with fidelity.

#### Characteristics of the intervention

*Perceived impact of initiative*. Some clinicians acknowledged the usefulness of the job aides and re-engagement forms in promoting standardized care, reducing variation among providers, and enhancing quality of care through proper documentation. Additionally, they believed that the job aides helped to make the correct care choices and improve overall healthcare delivery. The job aides were described by some participants as providing healthcare teams with a defined methodology to identify and implement clinical decisions.

*As I have said that SOP-9 [re-engagement initiative] acts as a guideline in terms of how to go about approaching and assisting the patient and also you go in-depth with the patient because it gives you a margin on what information you can ask from the patient. You get to [discuss] other challenges that you did not see before you use the SOP-9 [re-engagement initiative].*

***Clinician*.**

*Relative advantage*. The majority of healthcare providers perceived the re-engagement initiative as advantageous compared to existing processes. They noted its structured approach to assessing clinical conditions and the time elapsed since a missed appointment, as well as its ability to tailor follow-up management, including faster access to MMD and less-intensive DSD models, essential for achieving the last "95" in the "95-95-95" goals. The job aides empowered healthcare providers to target their clients’ specific challenges, providing a clear direction for improving service quality. Recognizing the initiative’s value and its potential benefits motivated them to put it into practice. These providers believed that the tools enhanced the quality of care they could offer to clients, aligning with broader national goals for re-engagement and retention in care. As one clinician shared:

*…for me it’s helpful and I do not see it as any addition to work. I think it impacts positively on the facility and my work because for me it makes me happy to see that people do come back and they remember that we are here to serve them.*

***Clinician***

While acknowledging certain advantages of the job aides, some clinicians perceived the re-engagement forms to be a disadvantage, not providing as much benefit compared to traditional working methods. They stated that the initiative, especially the re-engagement forms added minimal value and increased their workload, extending waiting times due to redundant recording of information, thus negatively impacting quality of care.

*…it adds more work, the form itself- it’s a problem because I spend more time with a patient that is being re-engaged. Although it does make everyone be managed in the same way, it also adds another aspect of workload. And we are saying we want people not to spend more time at the clinic but also with this paperwork we are making them stay longer-*

***Clinician*.**

#### Inner setting

*Leadership engagement*, *tension for change*, *and relative priority*. Engagement of the facility manager or another high-level representative was ensured before initiating the project. However, it was reported that scheduling time for leadership teams to meet, collaborate effectively, and communicate decisions with the facility staff posed logistical challenges. It was observed that managers were not actively involved in re-engagement services, creating an environment that was not conducive to the adoption and implementation of the initiative. While there was a sense that most facility leadership were generally supportive, they tended to take a hands-off approach, leaving clinicians responsible for implementation. This approach had a negative impact on the tension for change (the degree to which stakeholders perceive the current situation as intolerable or needing change) and the prioritization of the initiative for instance, some healthcare providers expressed uncertainty about how services would align with existing systems, as they felt that the new requirements contradicted the previous operational methods. Greater alignment could have been achieved if leadership had taken a more proactive role in driving the process.

The facilities that adopted the model and recorded positive outcomes were characterised by highly engaged leadership, highlighting the impact of engaged leadership in successful implementation.

*Compatibility*. The initiative’s compatibility was a complex issue, serving as both a facilitator and a barrier to implementation. It was discussed as aligning re-engagement efforts with various factors, including client needs, clinic goals, and existing workflows. One of the key facilitators identified, especially emphasized by non-clinical providers, was the potential for addressing treatment literacy. They highlighted that comprehensive counselling as part of the initiative had the potential to enhance clients’ understanding of treatment, ultimately leading to improved outcomes that aligned with the goals of the facility.

However, another group of participants raised concerns about the impact of the additional documentation (especially the re-engagement forms) on the client experience, including longer waiting times. The additional documentation requirements which were said not to be integrated into existing processes, coupled with a shortage of healthcare providers and high volume of clients, led to an increased workload and complication in client flow. Participants reported that the process of completing forms required considerable time and effort, which resulted in extended waiting times. Moreover, the documentation process was reported as a duplication, highlighting a lack of integration. According to one clinician:

*It’s a burden because if you have six patients that you normally see a day and they take about 18 minutes*, *with this form of engagement it can take an hour-*

*No, initially I started, I tried to*, *but I realised the distraction which came with it and how I was losing time at that particular time*, *and I stopped to say that this is just a repetition of the guidelines*.

***Clinician***

Participants emphasized the importance of maximizing existing tools and resources. They discouraged the introduction of additional forms for completion and instead advocated for the efficient utilization of the tools already at their disposal, despite these not being designed to support or document re-engagement processes. Additionally, some clinicians recommended that forms should be handled by other healthcare providers, such as data clerks, to distribute the workload and enhance efficiency. This approach would allow clinicians to prioritize direct client care. However, this suggestion overlooks the responsibility of clinicians in record completion, as well it the role of structured records in guiding clinical management.

*…have someone who is responsible for gathering or collecting for follow up purposes, whether it’s the retention officer or someone else because all these files end up with one person–the data capturer… find how the data capturer can help so that you can capture all the right information.*

***Clinician***

#### Readiness for implementation—Available resources and access to knowledge

*Training for the implementation*. All selected facilities were provided with training by clinician mentors who received train-the-trainer training. Reactions to training were mixed. Some healthcare providers wanted more information to manage and respond to client needs. They emphasized the need for ongoing training because the HIV environment constantly evolves, necessitating continuous updates to stay current.

*More training but not so much training but in-service training. I am not saying that we forget what we have been taught but we just need re-emphasis on things like things keep changing. We just to be kept up to date with everything.*

***Retention Counsellor***

In addition, the providers mentioned that there was limited training coverage in some instances. Most of the clinicians and data clerks receiving training, however some of the retention counsellors did not.

*Resource availability*. Staff shortages were also raised as a challenge to successful implementation. Participants described themselves as being overworked by the introduction of new initiatives.

## Discussion

Several elements of the CFIR framework were found to be relevant in this setting, as they played an important role in influencing the adoption, as well as potential success of the re-engagement initiative. The involvement of facility leadership played an important role in influencing implementation success. The majority of facilities had largely supportive but hands-off leadership. Facilities with actively engaged leadership experienced more successful implementation compared to those with supportive but less involved leadership. The study suggests that new interventions and practices are most effectively adopted when the intervention are supported by engaged leadership.

The relevance of "tension for change" in order for healthcare providers’ adoption of the intervention was obvious. Inadequate leadership driving tension for change and prioritization of the re-engagement initiative, negatively impacted adoption, aligning with previous literature emphasizing effective leadership [24–26]. Successful intervention implementation requires a comprehensive understanding of organizational change processes, including identifying decision-makers, addressing competing priorities, fostering shared agendas, and having strong leadership advocates [26, 27]. The perceived lack of leadership support left providers feeling solely responsible for implementation. Prior research suggests it is important to provide sensitization and feedback at various levels and stages, promoting upper-level ownership and support [27]. Given changing guidelines and priorities, clear communication and necessary training are crucial during the transition to prioritizing re-engagement [15, 26].

Another factor highlighted in this study relates to characteristics of individuals, specifically knowledge and beliefs about the intervention and self-efficacy. Most healthcare providers had a good understanding of the intervention’s objectives and the tools, but a few had negative attitudes and concerns. While some providers showed empathy for clients’ disengagement and understood the barriers involved, others displayed confrontational behaviours and patronizing attitudes, which made the implementation more challenging. These attitudes and levels of empathy influenced how well the intervention was adopted and its implementation. Negative attitudes and confrontational behaviours among staff members, particularly clinicians, were identified as factors that could discourage clients from maintaining re-engagement efforts. Previous studies in healthcare settings have highlighted the harmful effects of negative attitudes on client engagement and satisfaction [28]. This stresses the need for interventions that not only address clinical management but also promote a change in attitudes and behaviour and foster a client-centred organizational culture. Providers’ knowledge and beliefs about the specific intervention, in this case, re-engagement, can also play a crucial role in overcoming implementation challenges. We found that while some providers had problematic attitudes, many providers held positive beliefs, which encouraged them to adopt the intervention as an opportunity to improve client care. These health care providers could be encouraged and incentivised to act as champions for positive change [26, 27]. These challenges related to changing provider behaviour are not unique to re-engagement but extend to various areas of healthcare delivery, such as diabetes, cancer screening, and many others, highlighting the broader relevance of addressing provider behaviour change in healthcare interventions [26, 29, 30].

In addition to negative attitudes and confrontational behaviours, some clinicians viewed re-engagement efforts as disruptive. This negative perception of interventions aimed at improving client engagement has been observed in other studies [12, 28]. It highlights the importance of involving healthcare providers in the design, adaptation, and implementation of such interventions to address their concerns and misconceptions. Collaborative approaches that engage healthcare providers as partners in improving client engagement has shown to be more successful [31].

The alignment of the intervention with existing guidelines and facility goals also affected its adoption and sustainability. Non-alignment has been shown to adversely affect interventions, especially in resource-constrained public clinics [15, 24]. The intervention aligned with provider and facility goals including the UNAIDS 95-95-95 targets, and the desire to achieve quality services. While participants perceived the re-engagement initiative as generally beneficial, some providers found the additional form to be an unnecessary burden, affecting workflow. These findings emphasize the importance of tailored interventions that align with the capabilities and needs of healthcare providers and their clients, particularly in settings with high clinical demands and limited resources. Providers in our study recognized a relative advantage in the re-engagement job aides echoing findings from previous literature [15]. However, the re-engagement form was not well received by most providers as considered additional and unwarranted burden impacting workflow. These results are consistent with previous studies on guideline implementation [18, 24].

Our study had limitations as we did not quantify implementation outcomes, such as adoption, or fidelity. The study primarily relied on information gathered from the perspective of healthcare providers, potentially limiting the understanding of how leadership perceived the initiative. Due to time constraints and scheduling conflicts, only one out of the ten invited facility managers could be interviewed. Nevertheless, our study benefitted from employing a robust theoretical framework, the CFIR, which has broad applicability in implementation science and is increasingly utilized in low- and middle-income countries. Also, our study involved a strong coding and analytic approach with more than one analyst and the interview questions were open-ended, enabling participants to express their perspectives in-depth.

## Conclusion

Healthcare provider and system support for treatment re-engagement efforts made by people living with HIV is necessary for successful HIV programmes. To maximize the effectiveness of re-engagement initiatives, it is imperative to address healthcare providers’ perceptions and behaviours relating to missed appointments, disengagement, and re-engagement, towards a client-centred organisational culture. Involving facility-based healthcare providers in design and adaptation could contribute to this goal. Moreover, leadership commitment and active engagement plays a critical role. This involves ensuring that re-engagement initiatives are coherently integrated with the broader program objectives, such as achieving the 95-95-95 targets and delivering high-quality services.

## Supporting information

S1 File Re-engagement form. (DOCX)

S2 File Interview guides. (DOCX)

S3 File Transcript excerpts. (DOCX)
