# Supplementary figures and images for: Factors influencing the implementation of a guideline for re-engagement in HIV care in primary care settings in Johannesburg, South Africa: A qualitative study
Source: PLOS Glob Public Health. 2024 Oct 30;4(10):e0003765. doi: 10.1371/journal.pgph.0003765 (PMC11524482; doi:10.1371/journal.pgph.0003765)

**Re-engagement form**


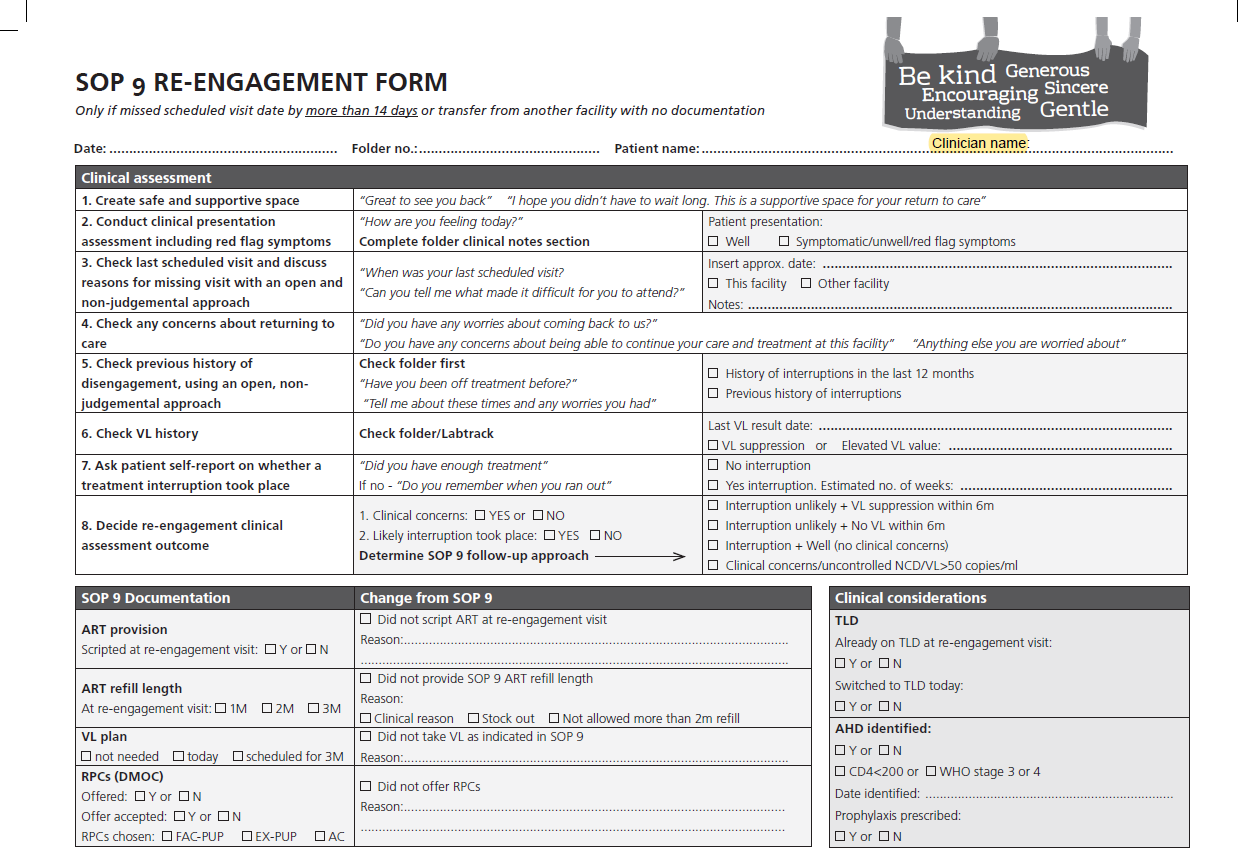

Supplement: S1 File — (DOCX) [file pgph.0003765.s001.docx]
