# Supplementary material for: Factors influencing the implementation of a guideline for re-engagement in HIV care in primary care settings in Johannesburg, South Africa: A qualitative study
Source: PLOS Glob Public Health. 2024 Oct 30;4(10):e0003765. doi: 10.1371/journal.pgph.0003765 (PMC11524482; doi:10.1371/journal.pgph.0003765)
Supplement: S2 File — (DOCX) [file pgph.0003765.s002.docx]

## **Interview guides**

**Interview guide – managers**

**Role of participants:**

1. Could you start by telling me a little about yourself and your experience with NAG SOP 9 on re-engagement?  **Please tell me about:**
   1. your role as a manager/supervisor
   2. your role as a facilitator of the intervention in the facility

**NAG SOP**

1. C**an you tell me about the** NAG SOP 9 impact on sustaining re-engagement/supporting clients to remain re-engaged when they come back at **this facility?**
   1. **How were re-engaging clients managed at your facility before this project?**
      1. **Were you aware of the NAG SOP 9 before this project?**
   2. **How are they managed since the project started?**
      1. **What are the key things that have changed, if any.**
   3. **In your opinion, how important is** NAG SOP 9 on re-engagement **at this facility?**
      1. **Does it help the client in any way – if so, how?**
      2. **Does is it support the facility with managing returning clients – if so, how?**
      3. **How helpful is it for guiding clinicians and counsellors?**
      4. **Does it change workload for staff, in what ways?**
   4. **Do you feel the SOP9 job aide tools met a need?**
   5. **How would you describe integration of the SOP with routine HIV care program activities?**
   6. **Do you think there are any remaining gaps in re-engagement in care at your facility?**
2. **What has been your own experience when working** with the NAG SOP 9 on re-engagement**?**
   1. Has it changed your routines for managing re-engaging clients?
   2. **Does the SOP approach fit well with normal practices in the facility?**
   3. **Does the project fit well with clients’ normal practice or expectations of their clinic experience?**
3. **What are some of the challenges with implementing the SOP?**
   1. **Prompts: Were/Are there any difficulties**
      1. **Identifying re-engaging clients**
      2. **Identifying clinicians to re-engage clients**
      3. **Using or following the job aides**
      4. **Completing the re-engagement forms**
      5. **Applying the algorithms in the job aides to manage re-engaging clients**
      6. We have noticed that some patients are not being managed according to their assessment e.g. Viral Load taken early or not given multi-month scripts. Can you provide any insights into why the job aide algorithms might not be being followed
4. Recommendations for improving the job aides to support SOP 9 implementation.
5. Are there any guidelines in the SOP9 that you would like to change?
6. Is there any guidance in the job aides that you would like to change?

**Prompt**: 3-month dispensing; when to do CD4s or viral loads; when to start DMOC or rescript a re-engaging client for DMOC, when to schedule a person for a return visit, when and how often to provide adherence counselling:

- - are there any areas in the re-engagement SOP and job aides where there is a disagreement between health professionals as to what is ideal?
  - Is there anything you would like to change regarding how clients are managed when re-engaging?
  - what do you like and dislike about the job aides
  - What do you like and dislike about the re-engagement form?

Overall, do you feel that structured management of re-engaging clients is something that should be prioritized within the ART program? Please explain your answer

**6. Training and supervision**

Did you receive any training on implementing the NAG SOP 9?

If you received training on implementing the NAG SOP 9: Re-engagement, what can you recall from that training?

Probe for:

- Good points on the training
- Expectations on the training, were the expectations met or not. If not, please explain.
- Do you think the information you learned in the NAG SOP 9: Re-engagement training was helpful? How? - Did the training help you in the daily routine of caring for clients? In what way? If not, what would have helped?
- Did the training provide a clear background/rationale for why SOP9 was being implemented at your facility for re-engaging patients?
  - Did you leave the training understanding the objectives/expected benefits of implementing SOP9?
- Improvement on the training
- Is there sufficient ongoing support and training? How useful were the supervision visits from your supervisor?

Do you want to add anything?

**Thank you!!**

1. **Interview guide – Implementers.**

| **Interviewer ID** |  |
| --- | --- |
| **Date of Interview** |  |
| **Region** |  |
| **Clinic Name** |  |
| **Interviewee Consented** |  |

**1. Role of participants:**

- Could you start by telling me a little about yourself and any role you play with clients who are late for appointment, or disengage from care or come back to care
- When did you start working at this facility? Years of experience as a clinician/ counsellor/pharmacist/ admin clerk? In HIV care? What motivates you to work in HIV Care?
- Could you tell me about your experiences with clients disengaging and those who do re-engage?

Probe for:

- Opinions about clients who disengage from care
- Stereotypes/assumptions

**2. General questions about Re-engagement:**

- What are your thoughts on clients who disengage from care?

Prompts:

- Less/more clients disengaging – is it a big/increasing problem?
- Do people come back? Why?
- How do you see your role in managing a person who has returned?
- How does disengagement/re-engagement impact the facility and your work.

**3. Training and supervision**

Did you receive any training on implementing the NAG SOP 9?

If you received training on NAG SOP 9: Re-engagement, what can you recall from that training?

Probe for:

- Good points on the training
- Expectations on the training, were the expectations met or not. If not, please explain.
- Do you think the information you learned in the NAG SOP 9: Re-engagement training was helpful? How? - Did the training help you in the daily routine of caring for clients? In what way? If not, what would have helped?
- Improvement on the training
- Is there sufficient ongoing support and training? How useful were the supervision visits from your supervisor? Did you have any chance to go through the tool with your supervisor

**4. Experience implementing NAG SOP 9: Re-engagement**

Have you been involved in working with people who have come back after missing an appointment or after a period out of care? How?

(Probe: was he/she involved in counselling, to what extent)

**How do you use NAG SOP 9 Re-engagement with your clients? *(admin clerk or similar)***

Probe for:

- - Can you tell me about the SOP 9 approach to re-engagement?
  - How do you understand your role in this?
  - What tools do you have to implement SOP 9?
  - Which tools do you use and how often do you use them?
  - How has your management of re-engaging clients changed since you were trained on SOP9 and given tools?
  - What is the challenging part of identifying a re-engaging client using the SOP?
  - Do you think you are the right person to take on this role or is there someone more appropriate?
  - **How do you use NAG SOP 9 Re-engagement with your clients? *(Counsellor/Retention counsellor)***

Probe for:

- - Can you tell me about the SOP 9 approach to re-engagement?
  - How do you understand your role in this?
  - What tools do you have to implement SOP 9?
  - Which tools do you use and how often do you use them?
  - Do you discuss with the client his/her reasons for missing visit? Yes or No and why?
  - How do you undertake a conversation about reasons for missing visit with your clients?
  - What makes you ask about reasons for missing visit?
  - When will you not talk about reasons for missing visit?
  - How has your management of re-engaging clients changed since you were trained on SOP9 and given tools?
  - What is the challenging part of identifying a re-engaging client using the SOP?
  - Do you think you are the right person to take on this role or is there someone more appropriate?
- **How do you use NAG SOP 9 Re-engagement with your clients? *(Pharmacy or Pharmacy Worker)***

Probe for:

- - Can you tell me about the SOP 9 approach to re-engagement?
  - How do you understand your role in this?
  - What tools do you have to implement SOP 9?
  - Which tools do you use and how often do you use them?
  - How has your management of re-engaging clients changed since you were trained on SOP9 and given tools?
  - What is the challenging part of identifying a re-engaging client using the SOP?
  - Do you think you are the right person to take on this role or is there someone more appropriate?

**How do you use SOP with your clients? *(clinician)***

Probe for:

- - How has your management of re-engaging clients changed since you were trained on SOP9 and given tools?
  - What is your experience of conducting re-engagement clinical assessment? (challenges/facilitators)
  - What is your experience of being required to make the decision whether a client has interrupted treatment? (challenges/facilitators)
  - What is your experience of deciding which follow up approach is needed? (Interruption +well etc.) (challenges/facilitators)
  - What is your experience of implementing the management plan once you have chosen an approach? (challenges/facilitators)
  - Can you discuss what creates the difficulties in any of the areas above?
- As a clinician, what is your preference in the following situations?
- A client who is re-engaging today, when do you think it is best to take their viral load?
- A client who is well and needs to come back in 3 months’ time for a viral load: how many months of ART should they get at a time? Why?
- Integrating tool into standard of care **(All) – *(Interviewer to have job aides with them so that the participant can refer to the aide and show where there are issues)***
  1. Do you think this program (SOP9 and Job aides) relieves or adds burden to your current workload? Why? (prompts: more/less visits, less visits but more time commitment for each visit, more complexity for some, easier to manage everyone the same way)
     1. Knowing SOP9 now, how best can this approach where all people re-engaging in care are no longer all managed in the same way be fitted into the existing clinic systems and client flow?
  2. Any challenges that you think will be difficult to work around?
  3. Any suggestions for modifications to improve and make it more manageable?
- Perceived impact of the tool
  1. Do you think this SOP-9 approach (algorithm job aides) had a positive or negative impact on the clients? Why or why not?
     1. How is this different to before SOP-9 was implemented?
     2. What part of the SOP-9 approach or any other approach best helps a returning client to continue with their care once they have returned and not interrupt treatment in the future?
  2. Do you think this SOP-9 approach (re-engagement form) had a positive or negative impact on the clients? Why or why not?
     1. How is this different to before SOP-9 was implemented?
     2. What part of the SOP-9 approach or any other approach best helps a returning client to continue with their care once they have returned and not interrupt treatment in the future?
  3. After training did you make use of the job aides with every patient, with some patients, not at all – and can you explain why you did or did not use the job aides.
  4. Do you want to continue using this tool? Why or why not?
  5. Have there been any client responses to the new re-engagement approach
     1. Probe -positive, negative, please describe any positive or negative client interactions arising from using the SOP9

Do you want to add anything?

**Thank you!!**
