# Supplementary material for: Factors influencing the implementation of a guideline for re-engagement in HIV care in primary care settings in Johannesburg, South Africa: A qualitative study
Source: PLOS Glob Public Health. 2024 Oct 30;4(10):e0003765. doi: 10.1371/journal.pgph.0003765 (PMC11524482; doi:10.1371/journal.pgph.0003765)
Supplement: S3 File — (DOCX) [file pgph.0003765.s003.docx]

INTERVIEWER: What are your thoughts on clients who disengage from care?

RESPONDENT: Most of them when they come back to care are not in a good state, there are those that the retention counselor calls them back, and eventually they do come they will tell you the reasons for not coming to collect it because I was out of the province so I was collecting from that side, and we always never have the proof of that, but then most of the time out will find those with stories of challenges of being unable to come and get their medication and those that come back because they are now sick.

INTERVIEWER: Less/more clients disengaging – is it a big/increasing problem?

RESPONDENT: I think at first there we a lot of patients that were disengaging, until Sister D, created a system that if you are a defaulter she will re-book you and ask you to come in the following morning and not stand in the line they just go straight into her office and she will re-initiate them, that motivated more patient to come back to the treatment because they saw that they are getting special treatment. While others then have that mentality of saying this is not working for me anymore, then they stop defaulting from the treatment then start coming on time and stop.

INTERVIEWER: Do people come back? Why?

RESPONDENT: Yes, they do come back, they do. I guess there are different groups because there are those who are still in denial, and there are those who never got fair treatment, I mean by the clinicians or any of the staff, some might feel like they are being judged, and some are from the local area and they do not want to be seen, they fear stigma because here at the clinic we separate patients based on what they are here for, when we group them we have chronic, acute and family planning and within the chronic, we do not say HIV, hypertension we just group them all. So a lot of people just assume that once you go to chronic line automatically you think they are HIV and they are already judging you. But the majority do come back.

INTERVIEWER: How do you see your role in managing a person who has returned?

RESPONDENT: I try by all means not to treat them in any different form, I leave all the questions for the clinicians because as the first person they encounter, I should make them feel welcomed to the clinic. I just do my job as normal and for me, I do not change my attitude from patient to patient.

INTERVIEWER: How does disengagement/re-engagement impact the facility and your work?

RESPONDENT: I believe that it slows down our productivity because now we tend to take longer time to attend to that person as compared to the person that comes in their date because now you will find out that this person needed to take blood and they will not be attended and released within a short time. The sister or Retention counselor needs to find out what are their challenges and work on the solution, so instead of taking 15 mins, it can take 45 to an hour. So, it does slow down productivity and sometimes even cause chaos because now people become impatient and want to know why this patient is taking longer. And we have to lie now to protect that person and say we are short-staffed.

INTERVIEWER: Have you been involved in working with people who have come back after missing an appointment or after a period out of care? How?

RESPONDENT: Yes, my encounter with the disengaged patients is when they get to the facility, I have to retrieve their files and if their files are not with us then I know they need to go see the retention counselor.

INTERVIEWER: Can you tell me about the SOP 9 approach to re-engagement?

RESPONDENT: For us, it’s a white form that we were given to write for all the patients that have stopped taking the treatment for over 14 days

INTERVIEWER: How do you understand your role in this?

RESPONDENT: I am guessing is to see the number of defaulters that we have in this facility whether it is increasing or decreasing.

INTERVIEWER: What tools do you have to implement SOP 9?

RESPONDENT: we have a white form.

INTERVIEWER: Which tools do you use and how often do you use them?

RESPONDENT: white form, for now, they have paused it but it was meant to be a daily thing.

INTERVIEWER: How has your management of re-engaging clients changed since you were trained on SOP9 and given tools?

RESPONDENT: I think because it applies across, it’s a formal way of bookkeeping our clients and not missing any information.

INTERVIEWER: What is the challenging part of identifying a re-engaging client using the SOP?

RESPONDENT: sometimes I feel like it’s a duplicate, this form is like a register, and now I have to fill in the same information in two different places because this white form does not go into the file Lawrence comes back and takes them.

INTERVIEWER: Do you think you are the right person to take on this role or is there someone more appropriate?

RESPONDENT: Yes, I am the right person. I have been here for almost five years and my colleague is fairly new.

INTERVIEWER: Do you think this program (SOP9 and Job aides) relieves or adds a burden to your current workload? Why?

RESPONDENT: it adds more work, the form its self-it’s a problem because I spend more time with a patient that is being re-engaged. Although it does make everyone be managed in the same way, it also adds another aspect of workload. And we are saying we want people not to spend more time at the clinic but also with this paperwork we are making them stay longer.

INTERVIEWER: Knowing SOP9 now, how best can this approach where all people re-engaging in care are no longer all managed in the same way be fitted into the existing clinic systems and client flow?

RESPONDENT: When we patients come here in the morning we collect all their cards and then we sort them out with their dates, and if you have missed the date we put your card together, so what we can do is that we can one clinician who deals with defaulters so that those who are coming back to the treatment cannot affect those that have not stopped taking their treatment. There is a sister here that works with PrEP and sometimes she is free and maybe she can deal with the defaulters I think that will lessen the burden of load that we are currently facing. Because the same sister who attends to the patients who are on time still has to see the defaulters and that deprives the service to be better and more efficient.

INTERVIEWER: Any challenges that you think will be difficult to work around?

RESPONDENT: I do not foresee any challenge with that.

INTERVIEWER: Any suggestions for modifications to improve and make it more manageable?

RESPONDENT: When we patients come here in the morning we collect all their cards and then we sort them out with their dates, and if you have missed the date we put your card together, so what we can do is that we can one clinician who deals with defaulters so that those who are coming back to the treatment cannot affect those that have not stopped taking their treatment. There is a sister here that works with PrEP and sometimes she is free and maybe she can deal with the defaulters I think that will lessen the burden of load that we are currently facing. Because the same sister who attends to the patients who are on time still has to see the defaulters and that deprives the service to be better and more efficient.

INTERVIEWER: Do you think this SOP-9 approach (algorithm job aides) had a positive or negative impact on the clients? Why or why not?

RESPONDENT: For me, it’s the same because we are still operating in the same way it’s just that now it’s a re-booking system, but the clients do not notice anything

INTERVIEWER: How is this different from before SOP-9 was implemented?

RESPONDENT: If I was to mention the change it will be time-consuming the implementation of SOP-9, because of what we used to do previously with the assistance of health promoters they will notify them by saying if you know that it is not your date today to collect you will be attended last and we will consider first that are coming on their correct date, and they were given options whether to come the following morning or wait until they are assisted at the end. So, SOP-9 has now forced us to squeeze those people in.

INTERVIEWER: What part of the SOP-9 approach, or any other approach best helps a returning client to continue with their care once they have returned and not interrupt treatment in the future?

RESPONDENT: I’m just guessing that maybe the conversation that the patient has with the retention counselor could be the one that motivates the patient to come back to treatment.

INTERVIEWER: Have you been involved in working with people who have come back after missing an appointment or after a period out of care? How? (Probe: was he/she involved in counselling, to what extent)

RESPONDENT: as I have mentioned that yes when they come back before they go to the clinician, they start with me I counsel them provide solutions to their challenges then they see a clinician.

INTERVIEWER: How do you use NAG SOP 9 Re-engagement with your clients? (Counsellor/Retention counsellor) for me, this is the form that I’m using for re-engagement, not the SOP 9 one. This one is even better than the one we used to use.

INTERVIEWER: What tools do you have to implement SOP 9?

It’s only this form, which is not SOP9 (I took the sample of the form)

INTERVIEWER: Which tools do you use and how often do you use them?

RESPONDENT: I use this tool every day, especially when attending the disengaged patients. If it’s a welcome back I fill in where it says welcome back and there are a lot of things that I fill in as you can see.

INTERVIEWER: Do you discuss with the client his/her reasons for missing the visit? Yes or No and why?

RESPONDENT: Yes, I do discuss with the client, because I want to find out where is the problem and to probe some of the things to get a clear picture so that they do not continue with them. So some will say that at school their teacher was teaching them about this and that and they want to find out if it’s true or not.

INTERVIEWER: How do you undertake a conversation about reasons for missing visits with your clients?

RESPONDENT: I first find out how the patient feels about taking the medication and I ask them why they missed the date almost all of them are in school so sometimes they will tell me that they were in school and didn’t get a chance to come to the clinic, so I will then tell them to come over Saturday if they see that they will not make it on their dates.

INTERVIEWER: What makes you ask about the reasons for missing a visit?

RESPONDENT: so that I can see if the problem is gib or small and what contributes to the decision of missing the date.

INTERVIEWER: When will you not talk about the reasons for missing the visit?

RESPONDENT: no there is no time.

INTERVIEWER: How has your management of re-engaging clients changed since you were trained on SOP9 and given tools?

RESPONDENT: not trained!

INTERVIEWER: What is the challenging part of identifying a re-engaging client using the SOP?

RESPONDENT: I do not have challenges in identifying a re-engagement patient however, one of the challenges though is that once they miss an appointment you need to follow up with them about why then they will make an excuse of school and all that, which leads to defaulting and missing an appointment secondly, another issue that comes out it’s the stories, they have a lot of stories so we even have a social worker on site so that sometimes we book them for the social worker. for example, a 16-year-old just left home and told them that she needs some fresh air and if she’s not home it’s not easy for them to take the medication the parent came here to report that she is gone and she took her clothes but left the pills. And another challenge again is that they do not have cell phones so it becomes hard sometimes to trace them.

INTERVIEWER: Do you think you are the right person to take on this role or is there someone more appropriate?

RESPONDENT: yes, I think so.

Integrating tool into the standard of care (All) – (Interviewer to have job aides with them so that the participant can refer to the aide and show where there are issues) this section was changed to fit the form that the Retention officer has been referring to for re-engaging the client

INTERVIEWER: Do you think this program (SOP9 and Job aides) relieves or adds a burden to your current workload? Why? (Prompts: more/less visits, less visits but more time commitment for each visit, more complexity for some, easier to manage everyone the same way)

RESPONDENT: no, it doesn’t add more jobs with the form that I use its easy for me. I don’t know about the SOP9 form. This form I use this until the patient is suppressed.

INTERVIEWER: Do you want to continue using this tool? Why or why not?

RESPONDENT: I would like to continue using this one because it’s straightforward.

INTERVIEWER: Since you have been part of SOP9, do you think there are more clients coming back to treatment or not?

RESPONDENT: I think its 50/50 because we still have those who will still have to trace, if we didn’t have to trace anyone then I would say that they are coming back in numbers.

INTERVIEWER: So, you say its 50/50 because the is still tracing that is being done?

RESPONDENT: Yes.

INTERVIEWER: Okay, and then when you look at your role as a nurse, for someone who is coming back late for their treatment, how do you see your role?

RESPONDENT: My role is just to give adherence. My role is to advise on honoring their appointments.

INTERVIEWER: Do you think your role is important ?

RESPONDENT: It is important even though they don’t take it as important as they patient themselves. because remember when someone wants to be late they will be late which is being ignorant on their part. They need to take responsibility of their

INTERVIEWER: So, because of their its decision whether they take treatment on time or not but because you give adherence you see your role as important?

RESPONDENT: Yes.

INTERVIEWER: Okay, in general how do you see people who have stopped taking their treatment or those who are coming back to taking their treatment, how does that impact this facility, lets start with those who have stopped taking their treatment?

RESPONDENT: Remember with Phuthuma there are those who are we have retained in care and there are those who are coming back to care, so it affects us greatly because it will look like we are not working, it does affect us in a negative way because they will say nurses at Imbalenhle or tracers and whoever is involved in taking care of the patients, it gives us a negative impavt. Remember on Phuthuma there are scores they use were they have green, that means we will remain on green forever because the is nothing that you can do.

INTERVIEWER: And those are the ones who are not coming to take their treatment?

RESPONDENT: Yes, because remember on TIER they appear as defaulters or lost to follow up dependinfg on they have stopped taking treatment, so it gives us a negative image.

INTERVIEWER: And those who are coming back treatment, how does that impact this facility?

RESPONDENT: We wil have a grat improvement because remember the Data capturers do update that a certain patient came, so its minus one defaulter, it’s a positive thing.

INTERVIEWER: And looking a SOP9 training, did you receive training on SOP9?

RESPONDENT: Yes, I did.

INTERVIEWER: And from that training what is it that you can recall, what do you remember from that training?

RESPONDENT: That the is a green… the is four…. What do I call them? The is on ewho is late but is not disengaged and the is one who is disengaged, the is one who is ill and the is one who is well.

INTERVIEWER: Okay, that is what you can recall, obviously when you go to training, some people go with expectation. Some people go to training with expectations about what they would like to achive when they go to training and others will see during the training. Where your expectations met when you attended that training?

RESPONDENT: I didn’t have any because I didn’t know what SOP9 was, I am part of the group that says we will see during training.

INTERVIEWER: Okay but when you left training, did you leave the training room understanding SOP9?

RESPONDENT: Not hundred percent but I understood, we only found out when we started doing it that we don’t do less than 14 days because we were doing everyone that was late.

INTERVIEWER: And do you think the information that you got from that training was it helpful?

RESPONDENT: It was.

INTERVIEWER: How was that helpful?

RESPONDENT: Remember now we are able to handle late patients.

INTERVIEEWER: Okay and does it help in the daily routine?

RESPONDENT: Initially it came as a burden becausewe were already burden because we already had oone nurse doing all those things but it became better when we had another nurse doing it. Its only now that we are used to it and we have a bit of staff members.

INTERVIEWER: Okay, is the sufficient training or ongoing training or they just trained you once and that was it?

RESPONDENT: They only trained us once and that was it.

INTERVIEWER: And do you think if the training was ongoing, do you think that would be helpful or maybe coming back and do a refresher training with you guys, do you think that will help?

RESPONDENT: It would but they have to do it practically, maybe if they can do it on the practical side like we have a late patient and then they do it practical, because in some facilities are like us before they are still doing the 0 to under 14 days.

INTERVIEWER: Okay, so before you use to treat some one if they come in the facility a day late ater their appointment date you would treat that person as a late patient?

RESPONDENT: Yes.

INTERVIEWER: Okay but now you say…..

RESPONDENT: 14 days and above.

INTERVIEWER: Okay, your role is a clinician and you also deal with late appointments in SOP9?

RESPONDENT: I do everything.

INTERVIEWER: But under SOP9 you deal with late appointments?

RESPONDENT: Yes, I don’t deal with the disengaged but if the nurse who deals with the disengaged is not there then everyone does it.

INTERVIEWER: Okay, so everyone does that if the other one is not here but when everyone is here then you focus on the lte appointments and she focuses on disengaged?

RESPONDENT: Yes.

INTERVIEWER: Lets look at the questions specifically for the clinician and we will also look at the scenario were the clinician who deal with the disengaged is not here at the facility. When you look at the management of the re-engaging client, how has that changed since you have been trained on the SOP9?

RESPONDENT: We were taking viral loads when they were coming back but now we take them after three momths that is what has changed.

INTERVIEWER: Oh before you were taking viral loads when they were coming to rhe facility?

RESPONDENT: We would take baseline and viral load when they come back, so now we are only taking some baseline bloods when they come and we only take viral loads after three months.

INTERVIEWER: Okay and then your experience in terms of someone who is re-engaging, lets look at the challenges for someone who is re-engaging?

RESPONDENT: The challenges how in what way?

INTERVIEWER: Things you come across daily?

RESPONDENT: If there are challenges it would be maybe the person is sick then we would have to exclude some of the opportunistic illnesses then the person wont re-engage on that day.

INTERVIEWER: For example if they have TB?

RESPONDENT: Yes, maybe they are coughing then we would not re-engaage the person on that day.

INTERVIEWER: Okay, besides that challenge, are the any other challenges?

RESPONDENT: Besides that there are no other challenges. Because we always had enough treatment and we always give out treatment.

INTERVIEWER: Okay, and then what is the experience required to be able to make a decision on whether the client has interrupted treatment?

RESPONDENT: Remembe with INH they are allowed to interrupted if its three months they continue, so it has always been like that with INH, os I assume that we already had a basis on that they have bene disengaged for that long you continue with treatment and if its more than 3 months then you start over again.

INTERVIEWER: So, basically its about looking at the time frame?

RESPONDENT: Yes the time frame on how long this person has not bene on treatment.

INTERVIEWER: Okay, as a clinician what is your preference in the following situation, so, I have two situations, a client who is re-engaging today, when do you think is best to take their viral load?

RESPONDENT: Three months later because that patient has not been taking treatment but also we can take on the same day to have a baseline for three months earlier and now it will be that virall load just to see if they have been taking treatment post re-engagement.

INTERVIEWER: Okay, you say it depends but mostly its three months later.

RESPONDENT: Especially for those who have disengaged for a longer period.

INTERVIEWER: Okay, a second situation a client who is well and needs to come back in three times, how many months of ART should they receive at a time?

RESPONDENT: Three months. Why bring them back? If the is nothing that you are going to do. Its up to them whether they take the treatment or they disengage again.

INTERVIEWER: Okay if they are well and you give them treatment for three months they can come back after three and the decision will be upon them whether they take the treatment or not?

RESPONDENT: Yes. Cause if you say I give you one month of treatment and they have to come back, its up to them if they come back or not.

INTERVIEWER: So, do it happen that someone who is suppose to take three months of treatment and they ask to take for one month and they will come back the following month for refill?

RESPONDENT: No it doesn’t happen.

INTERVIEWER: Okay, these questions apply to everyone whether a clinician or RC. Do you think SOP9 releaves or adds a burden to your current work load?

RESPONDENT: It adds.

INTERVIEWER: Why do you think so?

RESPONDENT: It adds a burden because the is a bunch of extra paper work that we need to complete as well, the tick register, the patient file and that.So, it makes it more work.

INTERVIEWER: Okay, does it make the patient to have more visits at the clinic or less?

RESPONDENT: Our patients get three months its not only the SOP9 patients. Its still the same.

INTERVIEWER: Its still the same the only thing is that its more paper work for you?

RESPONDENT: Yes.

INTERVIEWER: Okay, looking at SOP9 right now, how best can we implement SOP9 at this facility?

RESPONDENT: Ptient that have disengaged they shouldn’t be given special treatment because they will all disengage because they now know that when you disengage we will treat them in another way. They should be on the same stream as everyone and then when they get to the room then you fill out the SOP9 form.

INTERVIEWER: That is the best way?

RESPONDENT: Yes because when you give them their own room, we are going to have problems.

INTERVIEWER: That is how we should do it?

RESPONDENT: Yes it should be integrated to the servifes that are already been offered at the facility.

INTERVIEWER: Okay and then do you think there are any challenges that would be difficult to work around?

RESPONDENT: With SOP9?

INTERVIEWER: Yes.

RESPONDENT: Not really. We can workm with the ones we have.

INTERVIEWER: And how can we improve SOP9?

RESPONDENT: Like I said they shouldn’t be given special treatment because they are going to disengage. The patients even though they don’t tell you they see that those ones who have disengaged they have their own room, so it will become a struggle.

INTERVIEWR: And looking at SOP9, SOP9 approach do you think it has a positive or negative impact on clients?

RESPONDENT: Both. Because others don’t want to be excluded in saying okay these are SOP9 patients because that brings back the stgma thing. If it accommodate all chronic patients not just HIV patients because these others ones eee that the is a specific room for others, so it should accommodate everyone at the clinic.

INTERVIEWER: Okay, that is a negative impact that you have mentioned.

RESPONDENT: Yes because they feel that stigmatized because you have to go to a certain room unlike following the queue like everyone else.

INTERVIEWER: Okay, what is a positive impact?

RESPONDENT: I haven’t seen it, I think we are nit there yet were we can say patients who came back and re-engaged there are adhering. We are not the yet to say what are the positives.

INTERVIEWER: Okay, alright what paty of SOP9 approach best helps in retaining the client in care once they have returned and they don’t interrupt treatment?

RESPONDENT: We can not guarantee a person coming back for life, remember with SOP9 the is adherence, adherence is voluntarly, if someone wants to go for adherence you let them go for adherence and if they don’t want you don’t force them. I think it was compulsory that they should go for adherence counseling then they would understand what is going on with their lives.

INTERVIEWER: Do you think the SOP9 re-engagment form, has it had a positive or negative impact?

RESPONDENT: People don’t want to be asked where they come fro, so thy don’t want to be asked. It’s a challenge.

INTERVIEWER: So, you can say it has had a negative impact?

RESPONDENT: Yes. I don’t know why that is there maybe they wanted to gather reasons why people don’t come back or come back?

INTERVIEWER: Could you tell me about your experiences with clients disengaging and those who do re-engage?

RESPONDENT: over my experience, there are very few who come back sick, some come back because they feel that they have been not taking the treatment for a long time now and they are not feeling strong enough. But I have not encountered those that are very sick. Another problem is that they have not disclosed this to their partners and the people they are living with so sometimes it’s not easy to take their medication freely and to come to the clinic and they end up defaulting from the treatment. So I also teach them and try to find out how is the relationship with the partner since the person is not comfortable disclosing. Maybe they will respond by saying that their relationship is okay but they have not gained the strength to disclose to them, they are not free enough. And maybe I will continue probing and ask what the problem is with not taking the pills. They will then respond by saying sometimes I missed the time because we are together and I cannot take them and I will then teach them other ways of managing time for the treatment and tell them that if they continue like that they will end up opening a gap that will make the virus grow and I will then ask them what she can do better that will help him/her to continue drinking their medication? If the patient does not have an idea, I then ask them to bring their partner and ask them that we have asked them to visit the clinic, for example, if the patient came to the clinic because of STI, it becomes easy to lure the other partner to the clinic. That is when I tip them to come to me straight when they come back so that I can ask someone to test them both and we aim to get the other partner. Some will come clean and say I am not working, and this person supports me so if this person leaves who is going to support me? So that is how some of the patients are traced back to care.

INTERVIEWER: What are your thoughts on clients who disengage from care?

RESPONDENT: from the treatment, there are a lot of people who seem like they are defaulted but only to find that they have not defaulted from the treatment it’s only that they did not come on the date that was given to them. Sometimes they stay for a very long time without coming to the clinic and when they finally come the sister will ask them to come back the following day when you ask them why they were not taking the treatment you will find out that they still had the treatment. So sometimes because they have extra that makes them not come on the date that was given to them. So sometimes they do complain that when they come to the facility to fetch the treatment the lines are slow to move, and they become demotivated when they think about coming to the clinic on the required date.

INTERVIEWER: Do people come back? Why?

RESPONDENT: yes, they do come back, and they come back sometimes without being traced.

INTERVIEWER: How do you see your role in managing a person who has returned?

RESPONDENT: my role is important because others ended up being suppressed through the discussions that we have and motivation and teachings. As retention counselors, we have their study the disengaged clients, the high Viral load, and the vulnerable ones like taxi drivers because they do not want to wait for the longest time because of their work. So those become suppressed because they come straight to us when it’s their date, they do not stand in the lines because they need to quickly go back to work, so as truck drivers when they come back after they have missed the date the nurses do not speak nicely to them so we teach them that if you see that you are going to leave please come and tell us so that we can give you more treatment, and if they finished while you are there just go to the nearest clinic with your container.

INTERVIEWER: How does disengagement/re-engagement impact the facility and your work?

RESPONDENT: on the facility level I think it does not affect them because we do want people to come back to the treatment but also the nurses have this attitude of treating defaulters otherwise and it makes it difficult to make people understand their behavior when they come to us.

INTERVIEWER: Did you receive any training on implementing the NAG SOP 9?

RESPONDENT: yes, I did attend the training that was taking place in Bophelong

INTERVIEWER: If you received training on NAG SOP 9: Re-engagement, what can you recall from that training?

RESPONDENT: what I remember is that it’s for people who have missed their date for 3 weeks, so when you are probing with the patient, some have missed for a day or two, so it’s a matter of reminding them about the importance of taking medication on time and every day. And for those who have missed up to 3 weeks, you ask them their reasons and tell them about the benefits of taking the treatment and also emphasize that if you have missed the date do not stay home for a long time without coming back for your treatment.

INTERVIEWER: Expectations on the training, were the expectations met or not? If not, please explain.

RESPONDENT: when I attended the training, I didn’t even understand what was it about until I got there, they told us what it was SOP-9 and it was clear then

INTERVIEWER: Do you think the information you learned in the NAG SOP 9: Re-engagement training was helpful? How? - Did the training help you in the daily routine of caring for clients? In what way? If not, what would have helped?

RESPONDENT: yes, it was helpful because it has guidelines on what you need to teach a patient before you transfer the patient to the clinician.

INTERVIEWER: Improvement in the training. Is there sufficient ongoing support and training?

RESPONDENT: I think in terms of the support there is, but I also think that it’s simple and straightforward. Yes, and sometimes there are small books they gave us, and they are important. You can just give them to look at while waiting and sometimes some are illiterate sometimes, they have challenges because they are in English, so we try to make it practical for them because they understand it better. After all, we take the 3 pills and explain to them that red means your virus is still a lot in your body, and if black in the immune system it means its opening up, and then if you start the treatment and you take it well, the red will start changing into black, and if you continue taking the treatment well its ends up not showing the red that much. So we explain that and tell them to continue taking the treatment and condomize.

INTERVIEWER: How useful were the supervision visits from your supervisor? Did you have a chance to go through the tool with your supervisor

RESPONDENT: we have spoken about the tool when we came back from the training but for the counselor, it’s not the same as for the clinicians.

INTERVIEWER: Have you been involved in working with people who have come back after missing an appointment or after a period out of care? How?

RESPONDENT: Yes. I also counsel the patient as I have mentioned. Because these are the people that are not starting the treatment from the start, you have to ask what they have learned before about the treatment, you are doing that so that you can see if the patient still knows something about the treatment. so others it’s blank others have small information so that is where you start and remind them. Others remember that since I am on the treatment I have to condomize.

INTERVIEWER: Can you tell me about the SOP 9 approach to re-engagement?

RESPONDENT: I do not use the form, the form is for the sister and the receptionist,

INTERVIEWER: How do you understand your role in this?

RESPONDENT: what I understand is that I need to make sure that clients leave the facility with all understanding of the treatment

INTERVIEWER: What tools do you have to implement SOP 9?

RESPONDENT: there is a form for adherence that we use, it has a lot of questions and some of them ask the patient whether they know the motive of taking the treatment, and we fill in those answers received from them, also about the time that they need to set time to remind them about their treatment time. So we also teach the that if they miss the right time every day the virus has the potential of spreading out even though you are taking but time is important. So, the pills close the virus in and make it not spread over the body parts. We also emphasize that they should carry their medication so that they will be always on time. We also teach them to focus on themselves and their loved ones.

INTERVIEWER: Which tools do you use and how often do you use them?

RESPONDENT: I use the adherence, in the morning it is usually busy, so these days we were even saying that the number of them has dropped. The people we deal with as retention counselors are high viral load patients those are people that we have been seeing lately, the disengaged ones they are now scarce so I think as we keep teaching them they are correcting and changing their behavior.

INTERVIEWER: Do you discuss with the client his/her reasons for a missing visit? Yes or no and why?

RESPONDENT: what I do is that I introduce myself and tell them to be free because I am trying to assist so I then ask them the reasons that have led to not coming to the clinic. Some will say I have forgotten about my date, and I will tell them to write in their calendar, or maybe put a reminder in their cellphones.

INTERVIEWER: and why?

RESPONDENT: I think it’s because I need to know what I am dealing with so that we can work on the specific issue.

INTERVIEWER: How do you undertake a conversation about reasons for missing visit with your clients?

RESPONDENT: As I have mentioned that I introduce myself and let the patient also introduce themselves and start the conversation about what they know and find out the reasons they are taking the treatment and the importance of taking the treatment.

INTERVIEWER: What makes you ask about reasons for a missing visit?

RESPONDENT: because I do not have to assume, the patient needs to tell me all about it so that we can both find the solution to it.

INTERVIEWER: When will you not talk about the reasons for a missing visit?

RESPONDENT: there is no time I have to miss that part because I have to know the reasons for missing a date and find a solution so that it cannot happen again.

INTERVIEWER: How has your management of re-engaging clients changed since you were trained on SOP9 and given tools?

RESPONDENT: for me, I have gained more knowledge.

INTERVIEWER: What is the challenging part of identifying a re-engaging client using the SOP?

RESPONDENT: what happens here when they come in the morning all the patients will give us their cards so that we can find the files for them so those that work in the reception are the ones that find them because they check the date, so they will look for your file and if they do not find your file in the filling room that means you have missed the date. So, some will say they lost their cards because they are scared that they will be caught it’s not the first time coming to the clinic, so the database helps us a lot.

**KI:** I’m a clinician. Unfortunately, I don’t deal with them only, remember we don’t have a particular room for them, so we integrate them. So, with the number of patients you will be having for the day, you will have a surprise of I’m coming back. Meaning you have your 80 people for the day and then there is this one coming back, who is very much welcome.

Let me summarise this thing. The challenge for us was repetition of what is required, remember I still have to fill in the file and then there is this booklet which you want for your study – it was time consuming, that was a challenge.

Secondly, it would have been better if it was in a chart form rather than the paging, paging – imagine you have this patient and a queue out there. We re-engage you and you go back to treatment but now I have to confirm the SOP-09 which we previously discussed, not every clinician is familiar with what is expected. For me when I was answering, I thought every clinician is familiar, so they said it is for the purpose of giving guidance. Ok we have no problem of giving guidance, maybe in a simplified version, where you just point arrows, without the booklet. So, now you have a queue, you are taking bloods, you are filling forms, it gets congested. Hence, the receiving was not so welcoming. We are so overwhelmed, the last thing I can’t be writing forms and forms. I even suggested that how about when the data clerk is capturing, we put them aside there. If you are interested to evaluate if we are doing what is supposed to be done, you can go back to that.

For me to be going through that list, did you stop? The reality of our patients is that not all of them are going to be honest and tell you the truth. As much in a practical situation you want them to, it depends on the type of relationship you have with them. The type of care you give is determined by what comes up in that interview – what happened, why did you disappear. Most of our clients come from other countries, they didn’t stop because it’s hard for them to come to the clinic or what, I had a baby and culturally I had to go back home to stay with my in-laws or what not- we all do that. One thing I have realised with patients - between the work; I’m from KZN, I went home; I lost a job; I’m no longer staying her, the care is not going to be overly from the other side to say this is what you should be doing because some they will say that I didn’t stop, when they know very well they stopped. So, yes, it’s a good guideline, I don’t know how it can be modified in a simplified version. As much as you are saying for the clinician it’s just to remind them, because some are not familiar of what is expected – when to take bloods, how long have you defaulted and what not. The approach of it was a big complain.

And you are saying we must be put the person in a queue, sometimes it’s not possible, I have to ask the person because of this reason, today I’m fully booked, I will not be able to attend to you, can we arrange for another day. You know the clinic and the people, I cannot be promoting the one who was compliant come another day and this one comes whenever he wants and then I say, let’s re-engage you are first in line. You might be first in line but you are not booked for the day. I have people booked for today, who made sure they put everything aside to come on the date. Remember, I’m promoting life, so they should see the benefits of coming on my expected date. One thing this thing will promote, we will never know how to plan our day. There are days when you have 80 in a day, imagine if I can have 20 on top the 80 who come by 7am meaning I will have to send away the 20 booked on that day because I can’t accommodate them. It becomes a vicious cycle which we cannot control and for you the clinician it becomes more work. This one who came on their date and was not seen will not ask for another date to come back to you, they will say there is no point. So, we kind of reward those who honoured their dates, let’s make it as quick as possible then you go back to work. Those who are know they missed their appointments, ideally they can wait for us or if they cannot wait for us or you come back early tomorrow before I start the group for that day, and remember I will be knowing that you will be coming. I know the SOP-09 says we should prioritize them, on the ground it’s not practical. Remember we have cohorts, I group them according to these are taking bloods----and then you have this extra person, how do I accommodate that, with which manpower because you take bloods in the room, decant in the room, we script in the room – one room does everything. Imagine that workload for one person, that’s why we end up making so many errors because there is so much we are doing.

**Interviewer: So, taking me through what you exactly do in the room.**

KI: You are doing bloods, initiations, scripting for CCMDD, rescripting for CCMDD, seeing your normal chronic patients because you cannot divide them in different rooms- there is stigma and then re-initiations. Every chronic condition is seen in the same room, you determine this one is CCMDD, this one we deactivate CCMDD because of ABCD, this one is now pregnant, we take bloods for ANC, do you understand. It’s a lot of logistics. And now on top of these logistics, there is now this book, did the person stop. If I’m writing this thing on the file, why should I write it again. My time is pressured I don’t want to lie. Now I must take this very same information from this file to another file.

**Interviewer: In practical sense, do you think there should be a person who transfers this information from this document to this document?**

KI: Why does it have to be there because we only have one legal document, the file. From the clinician, it goes to the data capturer who classifies them according to this one is initiation, this one is re-initiation what not. If we have a section of re-engaged patients, this person should be familiar to say so that he keeps a record of them. Remember this person comes to me via the counsellor, so retention counsellor, myself and data capturer, we are repeating the same responsibility of capturing this same person. It’s repetition, for what.

**Interviewer: You feel this SOP-09 document is repetitive, more work, it’s not a legal document. The way you feel is that the general feeling of other clinicians?**

KI: Yes, it’s not a legal document and non-binding, it’s not going to promote the life of the patient we are not intervening in their life using this document. We are intervening using this file, which is what we use between us as clinicians for continuity and between adherence counsellors and where they record. So for me to know that you met so and so, and they defaulted because of dah dah it’s in the file and I should follow up using the file and answer whatever questions raised. Maybe you took bloods and we are evaluating if you are suppressing or not, it’s all in the file. But let me add that every the case that I have worked with the care relationship you have with the people matters, just as much as we have all these documents. In the room, it’s not the file, I listen the issues of the patient. Some of the problems, we will not be able to solve but we try.

There is only one legal document binding between me and the patient. Even if there are questions, something goes wrong, 6 months or 9 months later, the file is what I will use. I will not remember that there was a SOP-09 document. That’s the general feeling as clinicians, this file is what we work with. Even if the file is not there, I can’t use the SOP-09 to supplement this because this is not recognised. **(It is not recognised by who?)**. My employer – even in nursing practice, you are told there is only one source of truth, the legal document. They will even tell you that write everything in there, not somewhere else. I’m saying, can’t we use one document to source information and the different stakeholders who need information they can extract from this one. Let’s not create a parallel system, I’m writing the very same details in it. Ok they said some clinicians are not clear on what is expected, then let’s train the clinicians accordingly without bringing another textbook. Remember I have some already and now I’m carrying another in the room, then I’m paging through. Do you know the level of uncertainty which comes with you paging through the thing in the room, with a patient who feels I’m dying tomorrow. As soon as you detach your eyes even if they were about to tell you something that is right, they will stop. It's the blood form, it’s the file, it’s the laptop; it’s a lot of things that are distracting. That’s why I’m saying I have nothing against improving the care using the SOP-9 guidelines. Let’s have something simple which a clinician can stick it on the wall without perusing. The perusing is distracting. Then the bloods that I should to take I will forget to indicate that I’m to take CD4 count and VL because now I’m perusing through things. It’s a lot of commitment for one person, it is a lot to cover. Remember I said I’m seeing hypertension patients, so now even if I’m HIV positive, I have to be checked for BP. The anxiousness I have seen on some of the clinicians, it made me say realise as much a this is meant to be a good tool but somehow it’s like a compliance I don’t know what. Because now so and so comes, did you fill in? – I’m not gonna do something for the mere fact that I want to comply or make someone happy – No. Because now if you have not filled them in and then Tuesday comes and you remember oh so and so is coming to collect – are you gonna pull them all out.

The data capturers, it’s a team effort, the very same file which is circulating among us. If we say these reengaged patients after capturing them, bring them back because maybe we need to go through them or whatever, maybe evaluate them, it’s easier without us having to spend time on the admin part of it- it's unnecessary. Because we already have a lot to do, they have to separate people, do you know how hectic it get there at the reception? It’s just two guys. It’s not the only patients they see, they see everyone, the emergency they are also part of that. It’s like we are expecting the impossible from them, it ends up being a demotivating factor. It becomes a burden. You want us to improve the care through SOP-09 but it will be easier maybe if you just take the file and evaluate what we are doing and where we are not doing well, you indicate. It will be faster. If you are expecting me to write the things twice in the file, I’m not gonna do it, not because I don’t want but we don’t have the resources. I enter with the patient and I leave with the patient. To go and eat, you have to excuse yourself, they will be complaining. You will be apologizing 10 times but guys, guys because to them it’s frustrating as they would have been waiting in the queue for too long and then the clinician is flipping through pages, what kind of image are we giving. So we have nothing against the protocols of reporting and the guidelines but can we integrate it a way eeh package it nicely and in a scenario of someone crowded with work. It sounds good for someone in the office, thinking there are 5 people. If I had 5 people, trust me I would not have any issue.

**Interviewer: So, in a day can you give me the target number of patients you have?**

KI: I don’t have a particular target per se as long as I have cleared the line **(but in a typical day, on average you serve how many people?)**. Uuuh they are many, it depends. Normally we are 2, but now I’m 1 and I’m sitting here and the queue is waiting. If you are lucky you can serve 30 a day or 40, you are like, it’s a good day. Remember of those 30 you are not just but you are talking, mama how are you, how are you doing today? You are talking, taking bloods all those things. So you are doing work for 3 people and all in one room, with a number that is crazy. So you are doing much more than the physical because; one thing about HIV is more psychological than physical. If somebody understands the importance of ARVs, they are likely not to be a problem and if they miss that one on initiating, you will not win, you will not. That’s why they go missing because they don’t understand the point and purpose of treatment. But once you have won them, it’s much easier for them to adhere. So, from 35, 40, 45 and 80 on a worst day. Because sometimes the people who are supposed to be for 2 people, the person might not report for duty due to their personal problems. Those people are booked for the day must be cleared, all of them. By the time you knock off, you have to finish them that day, you cannot carry them over. It becomes a mission impossible with those who have missed and disappeared.

**Interviewer: You said one way you cater for those who have disengaged is to make them come the following day, are there any arrangement the facility makes for them?**

KI: If I’m not fully booked, I see them immediately. If I’m unable to see them immediately, I arrange for them to see me the next morning. Why I say in the morning it’s because while they are pulling files, there is a gap for me, for about an hour, which we normally use to tidy up. I can see those who are un-booked to be able to just come in without it

**Generally how does it make you feel to serve disengaged patients?**

The sad part is it is beyond you and me. It’s not nice to lose someone. Remember, the objective is to have a suppressed community so that we minimize transmission. So, now I have people who are disappearing for 2 years, what is happening out there and I know for sure they are condomizing. The transmission rate is not going down, I’m not sure if we are going to take it down. **(Why do you say so, just give me your experience).** People have these beliefs, mindsets and challenges that are beyond us. As much as I would want, some of the things they raise in this room. You see that block over there, when they are drunk on Friday, they shout that people on the other side can hear, “You cannot tell this and that you who is HIV positive. Do you think I’m gonna come back and collect my ARVs when I’m due. Everybody heard. It’s that kind of a culture, they call each other out with their worst scenarios. Meaning we haven’t won the community that being HIV positive is nothing to, so the stigma which existed in 2002 when I was a child hasn’t changed. And the stigma that made people survive then, the mindset, it makes HIV positive individuals to say I don’t care, I can’t make you care. I will give you all the tools and say this and this but if you have this man who is responsible for buying you food, clothing your kids, paying you rent, are you going to tell this man all of a sudden you have been in a relationship for 4 years, you are now positive with this last born. Are you going to tell this man. You know this man better than I do. Just as much as I recommend to you this and that, there are people who will say I can’t and you can see that they can’t. You can see the man is waiting outside, waiting for the lady to come out. He is here but not testing, no matter how much you persuade him, he says, no, no, I’m not testing. He is waiting outside to hear the results of the woman. You come out with a positive result, she is more scared of this man. How best can we do it. What I have seen they will comply when they are pregnant, as soon as they deliver, they stop coming. That is the pattern of everyday, as soon as they are pregnant they come back. You see now, it’s not just about HIV, it’s more of a psychological issue, it’s more of abuse, GBV; now it’s getting bigger. Until the owner of the problem is ready to face the challenge, there is nothing we can do. There are those if I come across them in the community because I walk around the community, they will say, sister I’m not coming. They think I’m gonna ask about, of which I’m not gonna ask that. When they see you, this person is looking for me. There are things which I don’t know what to do but we do our best. Will it be enough, I don’t know, I’m not sure.

The socio-economic environment is not supportive at all for all the efforts we are doing. Because all of the people here are very poor, in abusive marriages/relationships and abusive communities. The women from this neighbouring country, they will tell you from prevention, they will tell you I have to consult my husband. Where is the power then, we don’t have any. It’s not that clinicians don’t want to re-engage people, and that’s where we sometimes we get it the wrong way. We want people retained on treatment but sometimes the workload, the challenges and everything we come across it’s beyond you. There are people who will tell you I tested in 2013, since then I have never taken treatment and I’m not going to. They disappear, you send people to trace them and say come to the clinic, they might tell you because I’m at work, it’s difficult for me to come there.

It’s their choice, it’s my health, I should chose if I want it. If I’m already here and I feel like taking it this time around because I’m sick; most of them come when they are sick. They want go back- sometimes you tell them you need to go for TB testing because I’m suspecting TB and then ask them to come back. You are not even sure if they will come back. We are forever checking numbers and doing whatever but the reality is if this person believes that you didn’t give them this tablet because they believe the tablet cures everything, he might come back, it’s their choice guys. That is the message we should be taking back to the community that we are here but we also want you to do your part, we can’t force you. Some fetch the tablets but don’t take them, they put them in the house. Then we are busy saying the viral load, the viral load

**Interviewer: Have you been trained on SOP-09**

KI: No

**Interviewer: Would you want to be trained?**

KI: There is no harm. Remember we are modifying a tool here.

**Interviewer: Do you feel that you have enough ongoing support**

KI: No, we don’t have. Do you know how hard it is even to get a stapler. To ease the workload for this person, whom you are saying should go through your booklet that you are saying they must refer to which I still don’t understand as you already gave me the guidelines.

**Interviewer: Please take me through the process of re-engagement.**

KI: the patient comes from the reception, take out their file. Normally they will direct them from the reception to the retention counsellor. Before they come to me they would have gone via her because I need her to follow up on them. They go there and do their vitals but most, they would have brought the file to me and explain that there is a person who has missed by this much, and I ask how is the queue outside. Ha, it’s hectic, so I will come go out and see the patient and tell them is it possible for me to see you tomorrow between this time and this time because of 1234. Normally they understand, one thing I have realised about people is that when you engage with them they understand. Before they leave and say I will come tomorrow, they would have finished with the retention counsellor and by the time they come back tomorrow, it’s only to finish my part of things. The only challenge is the clinic that one person alone with that queue. If it’s not packed, they go to the retention counsellor, from her them come to the room, we take the bloods and re-engage. If there is need for decanting, we decant. But each case differs, it’s not a one blanket approach, that’s the thing. At the end of day we are not going to leave any individual.

With men if you sit them down and talk to them to say these are the benefits, this is what you are going to gain. They like being pampered and made special. The moment you say, you are not going to queue, you come straight to me, more problems as they will believe I’m not gonna queue. Most people have trouble with queuing, it’s the biggest challenge. Most men cannot cope with sitting in one space for the whole day waiting for the nurse to serve them, they can’t They will tell you I have to go to work, I have to do what what. It’s not about that, one thing I have learnt it’s not about that, it’s about their personal feelings of not wanting to be seen in the queue. These are things we have to iron out with them, you being asthmatic, hypertensive, you don’t know why you are sitting in the queue. Sit in the queue comfortably, you might gain something. Most men in general they don’t like it, it’s like shopping, show me a man who likes shopping. So, can you imagine this wife who is coming with a husband, both are coming to collect. We have diagnosed them both now, the wife complies but the husband don’t because as much as the wife can collect on behalf of the husband, there are certain visits he has to do. There are points when I have to do assessment on the patient. When the husband is due, he doesn’t come back. So, it’s not an issue that people are not being re-engaged but it’s because of the stigma, it’s still as high as 2000.

**Interviewer: With the SOP-09 guidelines, has the way you manage clients changed?**

KI: Maybe it’s because I’m new in the department, for me, nothing has changed. I have been in here for about one and half years but have been here for almost 3 years. The guidelines didn’t change, you just took out the guidelines – it’s the same guideline, it’s just that they are put in a nice brown book and goes down and down.

**Interviewer: how can the guidelines and processes be improved?**

KI: Have someone we stick on the wall, and have someone who is responsible for gathering or collecting for follow up purposes, whether it’s the retention officer or someone else because all these files end up with one person – the data capturer. They are the ones who gonna know this one HAS missed for 2 years or for 6 years. We need to have a better strategy of how best to accumulate those names and the follow up plans. Clinicians are going to do the follow up in the room, because when I do the notes I make a plan that you should do 123 next visit. I have already done that, the only thing is you want to see what follow up did I do for the patient because you were not inside the room. There should be somebody when they are capturing to say the next clinic date. Maybe we should have where they put so and so is one of the people who were re-engaged. There is no need for us to be accumulating books, different books because you will find that the they are not even filled; when I remember I will fill in the book but when I don’t remember then what happens but all the files end up with the data capturer.

For example, sometimes you find that a clinician rescripted for CCMDD but on the scripting dates are for 2 months and the issuing dates for treatment I indicated that I issued or 3 months. That tells that person it’s human error – they normally come back and say I see 123 because if you didn’t rescript, you even indicate that not rescripted. We are talking there with each other as a team, why do we need to go out and change these things. And now we want to create another system and you coming to check if there are any people indicated for that week. You are missing the vital information because all the information I have goes to the data capturer, find how the data capturer can help so that you can capture all the right information. He can provide all that information we initiated 5 today on TLD and then awaiting is 1.

**Interviewer: Any part of the SOP-09 approach which you think can be identified as best practice?**

KI: The very same approach that we have if it’s simplified and put in an easily accessible format, don’t just put in a format which can be accessed by a few. Remember we said some people are not familiar with the guidelines, maybe they are in my room, they should be able to continue with the work. Put it in a simplified manner, without the fidgeting in front of the patient. If it’s in front of me, I can’t miss it. Just like when you are initiating, if the person waits this much , from this wait, do this. The very same thing, have it in a simplified chart, and people use it to refer , we are not using it as a guideline but for reference, to remind myself don’t forget, don’t forget. We can’t create another book again, because I’m not gonna read it. If I already have the guidelines and I’m not reading it, do you think I’m gonna read it…No. Put it in a simplified manner.

Interviewer: Is the SOP-09 positively or negatively impacting clients?

KI: Negatively, one, although this clinician is already under pressure, Anova is coming, I’m supposed to have these names and saying we should comply, what do we do when we are pressured, we take it on those who are supposed to be made to feel welcome, whom we are supposed to be intervening, social, economic or whatever. Because now the concern is the document and not the patient. I’m not gonna pay attention to this patient, I’m gonna pay attention to this compliance to please the bosses or the supervisors. If I will be fidgeting and the patient was gonna tell you that my husband is beating me, I will stop the story because you are not giving me attention, you are busy uuuuuh with the papers. There is so much here, especially in the rooms. We are too busy in the rooms with papers for the sake of compliance and not worried about the people in front of us. Hence, I’m saying can we stop paging and sourcing information which we can get find in the file of the patients. We all have access to one document but you now want it to be divided to 10, why.

Interviewer: Did you make use of the job aides with patients?

KI: No, initially I started, I tried to but I realised the distraction which came with it and how I was losing time at that particular time and I stopped to say that this is just a repetition of the guidelines. I’m not sure, I might be wrong, if you are functioning in a specific room, let’s say I’m delegated to do ANC, it’s my responsibility to go back to the guidelines of ANC, it’s my responsibility to become familiar with. I can’t be paging through when the patient is here. Yes, we do refer, we have that allowance of referring, we are having those cases which are out of the ordinary but re-engaging a patient is not out of the ordinary. You cannot finish a day without re-engaging. If you are working in a way that you are trying your best, you should be knowing SOP-09, even though you don’t know it’s SOP-09 but you should know what is expected of you. If we know how to manage for example, high blood pressure and diabetes, the very same ART patients you should know, if this has happened, this is what should happen and this is my aid for referral not to say you are studying in the room. And we should support and lead people in a manner that they feel competent, competency is a very important thing. There are managers when they delegate to you that you are going to do ABCD, they should have evaluated that you are familiar. If you are not familiar, you should not go there. When I started there, I was not equipped but I had someone with me in the room coaching me. That’s the only thing we need to be doing, equipping people to be competent. Truly speaking, this thing is just an excuse that clinicians don’t know, how can you not know and what are we doing about it. So, we reinforcing people not knowing things and saying we gonna brings things, then we are supporting people wrongly, honestly. If as a clinician my supervisor audits my file and pick up those mistakes, they should highlight it as area she might need further training. Before we make these documents and make them big, we can send them in the rooms, you can see that she got it the right way here but here she went wrong, maybe we need to do this and that to improve her skills. I doubt there will be someone who will say I’m doing it correctly, you can’t tell me. People are doing things wrongly and because even if we pick up the things, instead of fixing the wrong, we go and develop documents. If I say I’m NIMART trained, I should know how to re-engage. But even if I don’t know and you pick it up as my supervisor and you leave me like that and you go and come back with a book, do you think I’m gonna read it.

Relations go beyond the patient and nurse, nurse-to nurse, colleague to colleague. Admin, open the doors to say come and ask me, sometimes they get confused with the terms. You cannot go angry because they will come to ask – explain, that’s the only way. Guys if you say 123, please it means ….. Even if you see that one who was supposed to take bloods didn’t take bloods, go and find out.

The problem with our type of approach in health when I see a problem it’s an opportunity to create another problem, hence we are complicating our own lives. We will never meet your targets and I won’t meet my targets and whatever we are trying to do out there, we will never meet it. Our problems are not going to be solved by documents, simplifying things for people if you realise that the person can’t get it right. People are willing to do it if they are capacitated.

I got three books when I came back from sick leave, what is the purpose of this, why are we doing this. Aaah they want it; who wants it, can’t we challenge the person so that we can simplify in a way that it works for us. I also want to know how many people I re-engaged, did they come back after that day.

INTERVIEWER: Could you tell me about your experiences with clients disengaging and those who do re-engage?

RESPONDENT: over my experience, there are very few who come back sick, some come back because they feel that they have been not taking the treatment for a long time now and they are not feeling strong enough. But I have not encountered those that are very sick. Another problem is that they have not disclosed this to their partners and the people they are living with so sometimes it’s not easy to take their medication freely and to come to the clinic and they end up defaulting from the treatment. So I also teach them and try to find out how is the relationship with the partner since the person is not comfortable disclosing. Maybe they will respond by saying that their relationship is okay but they have not gained the strength to disclose to them, they are not free enough. And maybe I will continue probing and ask what the problem is with not taking the pills. They will then respond by saying sometimes I missed the time because we are together and I cannot take them and I will then teach them other ways of managing time for the treatment and tell them that if they continue like that they will end up opening a gap that will make the virus grow and I will then ask them what she can do better that will help him/her to continue drinking their medication? If the patient does not have an idea, I then ask them to bring their partner and ask them that we have asked them to visit the clinic, for example, if the patient came to the clinic because of STI, it becomes easy to lure the other partner to the clinic. That is when I tip them to come to me straight when they come back so that I can ask someone to test them both and we aim to get the other partner. Some will come clean and say I am not working, and this person supports me so if this person leaves who is going to support me? So that is how some of the patients are traced back to care.

2. General questions about Re-engagement:

INTERVIEWER: What are your thoughts on clients who disengage from care?

RESPONDENT: from the treatment, there are a lot of people who seem like they are defaulted but only to find that they have not defaulted from the treatment it’s only that they did not come on the date that was given to them. Sometimes they stay for a very long time without coming to the clinic and when they finally come the sister will ask them to come back the following day when you ask them why they were not taking the treatment you will find out that they still had the treatment. So sometimes because they have extra that makes them not come on the date that was given to them. So sometimes they do complain that when they come to the facility to fetch the treatment the lines are slow to move, and they become demotivated when they think about coming to the clinic on the required date.

INTERVIEWER: Do people come back? Why?

RESPONDENT: yes, they do come back, and they come back sometimes without being traced.

INTERVIEWER: How do you see your role in managing a person who has returned?

RESPONDENT: my role is important because others ended up being suppressed through the discussions that we have and motivation and teachings. As retention counselors, we have their study the disengaged clients, the high Viral load, and the vulnerable ones like taxi drivers because they do not want to wait for the longest time because of their work. So those become suppressed because they come straight to us when it’s their date, they do not stand in the lines because they need to quickly go back to work, so as truck drivers when they come back after they have missed the date the nurses do not speak nicely to them so we teach them that if you see that you are going to leave please come and tell us so that we can give you more treatment, and if they finished while you are there just go to the nearest clinic with your container.

INTERVIEWER: How does disengagement/re-engagement impact the facility and your work?

RESPONDENT: on the facility level I think it does not affect them because we do want people to come back to the treatment but also the nurses have this attitude of treating defaulters otherwise and it makes it difficult to make people understand their behavior when they come to us.

3. Training and supervision

INTERVIEWER: Did you receive any training on implementing the NAG SOP 9?

RESPONDENT: yes, I did attend the training that was taking place in Bophelong

INTERVIEWER: If you received training on NAG SOP 9: Re-engagement, what can you recall from that training?

RESPONDENT: what I remember is that it’s for people who have missed their date for 3 weeks, so when you are probing with the patient, some have missed for a day or two, so it’s a matter of reminding them about the importance of taking medication on time and every day. And for those who have missed up to 3 weeks, you ask them their reasons and tell them about the benefits of taking the treatment and also emphasize that if you have missed the date do not stay home for a long time without coming back for your treatment.

INTERVIEWER: Expectations on the training, were the expectations met or not? If not, please explain.

RESPONDENT: when I attended the training, I didn’t even understand what was it about until I got there, they told us what it was SOP-9 and it was clear then

INTERVIEWER: Do you think the information you learned in the NAG SOP 9: Re-engagement training was helpful? How? - Did the training help you in the daily routine of caring for clients? In what way? If not, what would have helped?

RESPONDENT: yes, it was helpful because it has guidelines on what you need to teach a patient before you transfer the patient to the clinician.

INTERVIEWER: Improvement in the training. Is there sufficient ongoing support and training?

RESPONDENT: I think in terms of the support there is, but I also think that it’s simple and straightforward. Yes, and sometimes there are small books they gave us, and they are important. You can just give them to look at while waiting and sometimes some are illiterate sometimes, they have challenges because they are in English, so we try to make it practical for them because they understand it better. After all, we take the 3 pills and explain to them that red means your virus is still a lot in your body, and if black in the immune system it means its opening up, and then if you start the treatment and you take it well, the red will start changing into black, and if you continue taking the treatment well its ends up not showing the red that much. So we explain that and tell them to continue taking the treatment and condomize.

INTERVIEWER: How useful were the supervision visits from your supervisor? Did you have a chance to go through the tool with your supervisor

RESPONDENT: we have spoken about the tool when we came back from the training but for the counselor, it’s not the same as for the clinicians.

4. Experience implementing NAG SOP 9: Re-engagement

INTERVIEWER: Have you been involved in working with people who have come back after missing an appointment or after a period out of care? How?

RESPONDENT: Yes. I also counsel the patient as I have mentioned. Because these are the people that are not starting the treatment from the start, you have to ask what they have learned before about the treatment, you are doing that so that you can see if the patient still knows something about the treatment. so others it’s blank others have small information so that is where you start and remind them. Others remember that since I am on the treatment I have to condomize.

How do you use NAG SOP 9 Re-engagement with your clients? (Counsellor/Retention counselor)

INTERVIEWER: Can you tell me about the SOP 9 approach to re-engagement?

RESPONDENT: I do not use the form, the form is for the sister and the receptionist,

INTERVIEWER: How do you understand your role in this?

RESPONDENT: what I understand is that I need to make sure that clients leave the facility with all understanding of the treatment

INTERVIEWER: What tools do you have to implement SOP 9?

RESPONDENT: there is a form for adherence that we use, it has a lot of questions and some of them ask the patient whether they know the motive of taking the treatment, and we fill in those answers received from them, also about the time that they need to set time to remind them about their treatment time. So we also teach the that if they miss the right time every day the virus has the potential of spreading out even though you are taking but time is important. So, the pills close the virus in and make it not spread over the body parts. We also emphasize that they should carry their medication so that they will be always on time. We also teach them to focus on themselves and their loved ones.

INTERVIEWER: Which tools do you use and how often do you use them?

RESPONDENT: I use the adherence, in the morning it is usually busy, so these days we were even saying that the number of them has dropped. The people we deal with as retention counselors are high viral load patients those are people that we have been seeing lately, the disengaged ones they are now scarce so I think as we keep teaching them they are correcting and changing their behavior.

INTERVIEWER: Do you discuss with the client his/her reasons for a missing visit? Yes or no and why?

RESPONDENT: what I do is that I introduce myself and tell them to be free because I am trying to assist so I then ask them the reasons that have led to not coming to the clinic. Some will say I have forgotten about my date, and I will tell them to write in their calendar, or maybe put a reminder in their cellphones.

INTERVIEWER: and why?

RESPONDENT: I think it’s because I need to know what I am dealing with so that we can work on the specific issue.

INTERVIEWER: How do you undertake a conversation about reasons for missing visit with your clients?

RESPONDENT: As I have mentioned that I introduce myself and let the patient also introduce themselves and start the conversation about what they know and find out the reasons they are taking the treatment and the importance of taking the treatment.

INTERVIEWER: What makes you ask about reasons for a missing visit?

RESPONDENT: because I do not have to assume, the patient needs to tell me all about it so that we can both find the solution to it.

INTERVIEWER: When will you not talk about the reasons for a missing visit?

RESPONDENT: there is no time I have to miss that part because I have to know the reasons for missing a date and find a solution so that it cannot happen again.

INTERVIEWER: How has your management of re-engaging clients changed since you were trained on SOP9 and given tools?

RESPONDENT: for me, I have gained more knowledge.

INTERVIEWER: What is the challenging part of identifying a re-engaging client using the SOP?

RESPONDENT: what happens here when they come in the morning all the patients will give us their cards so that we can find the files for them so those that work in the reception are the ones that find them because they check the date, so they will look for your file and if they do not find your file in the filling room that means you have missed the date. So, some will say they lost their cards because they are scared that they will be caught it’s not the first time coming to the clinic, so the database helps us a lot.

INTERVIEWER: Do you think you are the right person to take on this role or is there someone more appropriate?

RESPONDENT: yes, I am the right person because the knowledge that I share with them helps them a lot. And without me as a retention counselor, the clinic will be losing so many patients.

INTERVIEWER: Do you think this program (SOP9 and Job aides) relieves or adds a burden to your current workload? Why? (prompts: more/fewer visits, fewer visits but more time commitment for each visit, more complexity for some, easier to manage everyone the same way)

RESPONDENT: responses are based on the form she uses not the SOP-9 FORM

It’s a lot of work, the retention on it owns it’s a lot of work because we even go to the redcap tool so some of the disengaged patients are not my caseload it’s for the facility but anyway, we do fill and assist everyone, so that when they check the files they could see that we have done the adherence. They sometimes come for inspection so when thy check that form they see that the work has been done on that patient because they say that if that form is empty it means that there was nothing that was done on that patient.

INTERVIEWER: Knowing SOP9 now, how best can this approach where all people re-engaging in care are no longer all managed in the same way be fitted into the existing clinic systems and client flow?

RESPONDENT: I think the form helps in a sense to trace what you have done with the patient, so I think it is helpful because everyone is taught the same thing.

INTERVIEWER: Any challenges that you think will be difficult to work around?

RESPONDENT: No.

INTERVIEWER: Any suggestions for modifications to improve and make it more manageable?

RESPONDENT: For now I think it’s fine.

INTERVIEWER: Do you think this SOP-9 approach (algorithm job aides) had a positive or negative impact on the clients? Why or why not?

RESPONDENT: to other clients, it’s difficult because they are in the rush now, I have to fill in this form finish it, and start another one and that person still needs to leave for work. So I always tell them that if they do not want me to waste their time they should not stop their treatment.

INTERVIEWER: How is this different from before SOP-9 was implemented?

RESPONDENT: back in the day we use to write on small papers, we did not have space where we write because they said that the file is for the sister to write, and we did not have space so now it’s good because I will just fill in that forms and place it back to the file. So, we tick and tick.

INTERVIEWER: What part of the SOP-9 approach or any other approach best helps a returning client to continue with their care once they have returned and not interrupt treatment in the future?

RESPONDENT: I think this one is easy because we tick and tick than write.

INTERVIEWER: Do you think this SOP-9 approach (re-engagement form) had a positive or negative impact on the clients? Why or why not?

RESPONDENT: positive impact because I have a guideline to make sure that all my clients receive the same information and I receive the same information in all of them.

INTERVIEWER: How is this different from before SOP-9 was implemented?

RESPONDENT: firstly it has the same information and I can be able to gather all the needed information.
